# Cocaine by-product detection with metal oxide semiconductor sensor arrays†

**DOI:** 10.1039/d0ra03687k

**Published:** 2020-08-04

**Authors:** Paula Tarttelin Hernández, Stephen M. V. Hailes, Ivan P. Parkin

**Affiliations:** Department of Health & Life Sciences Alison Gingell Building, Whitefriars St Coventry CV1 5FB UK ad0561@coventry.ac.uk; Department of Computer Science, University College of London 66-72 Gower Street London WC1E 6BT UK; Department of Chemistry, University College London 20 Gordon St London WC1H 0AJ UK

## Abstract

A range of n-type and p-type metal oxide semiconductor gas sensors based on SnO_2_ and Cr_2_O_3_ materials have been modified with zeolites H-ZSM-5, Na-A and H–Y to create a gas sensor array able to successfully detect a cocaine by-product, methyl benzoate, which is commonly targeted by detection dogs. Exposure to vapours was carried out with eleven sensors. Upon data analysis, four of these that offered promising qualities for detection were subsequently selected to understand whether machine learning methods would enable successful and accurate classification of gases. The capability of discrimination of the four sensor array was assessed against nine different vapours of interest; methyl benzoate, ethane, ethanol, nitrogen dioxide, ammonia, acetone, propane, butane, and toluene. When using the polykernel function (*C* = 200) in the Weka software – and just five seconds into the gas injection – the model was 94.1% accurate in successfully classifying the data. Although further work is necessary to bring the sensors to a standard of detection that is competitive with that of dogs, these results are very encouraging because they show the potential of metal oxide semiconductor sensors to rapidly detect a cocaine by-product in an inexpensive way.

## Abstract 2

Metal oxide semiconductor gas sensors based on SnO_2_ and Cr_2_O_3_ were modified with zeolites H-ZSM-5, Na-A and H–Y to create a gas sensor array to detect cocaine by-product, methyl benzoate. SVMs were later used with a 4 sensor array to classify 9 gases of interest.

## Graphical abstract

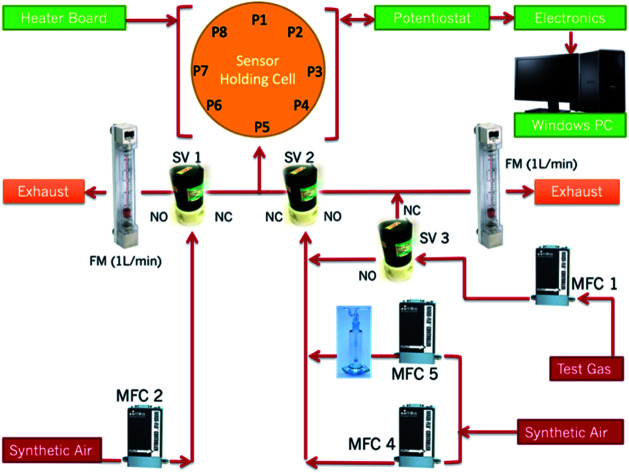

## Introduction

Metal oxide semiconductor (MOS) gas sensors have been heavily researched and proven to be suitable for a broad range of applications that are concerned with the detection of vapours relevant to environmental, health, safety and security-related fields.^1–5^ Although the feasibility of detecting drugs of abuse with solid state sensor technology has been highlighted in a number of studies, only one study has carried out explicit illicit drug testing with MOS devices.^6^ A more recent study revealed that a MOS gas sensor array could be used with a 92.5% accuracy to determine whether or not an individual had consumed cannabis, based on the different smells emanating from human skin.^7^ It is worth noting that other solid state gas sensor technology has been tried for the detection of illegal drugs such as ephedrine, tramadol, nalbuphine, agomelatine, methamphetamine, cocaine, and also for the detection of doping.^8–13^

Detection dogs are trained to target substances that have a detectable odour, which may be a substance other than the drug itself, such as a drug by-product.^14,15^ Indeed, drugs often have very low vapour pressures, which somewhat limits the chances of successful direct detection. A clear example of this is the detection of methyl benzoate – a by-product of cocaine – as opposed to cocaine, *per se*. Methyl benzoate has a sweet aroma and is targeted in canine detection as an indicator of the presence of cocaine (Fig. S1†). As highlighted in a previous manuscript by the authors,^16^ detecting drug markers in a way that mimics canine detection could prove particularly useful in security settings. It is worth noting that although methyl benzoate is also produced by snapdragon flowers, it is only at very low levels.^14^ Previous studies have shown that methyl benzoate is not the most significant contributor to the odour signature of the flowers. In fact, cocaine detection dogs will not alert to the snapdragon flower odour, as the odour profile has been found to be statistically different to that of cocaine.^14^

It is also worth bearing in mind that the illegal production of drugs may be carried out by inexperienced individuals that may not take the necessary precautions to ensure that products are safe.^17^ The lack of quality control and assurance in such illegal processes can mean that the presence of residual solvents may still be detectable both in drug samples and/or the environment in which they are synthesised.^18^ These solvents, which have been proven to be detectable with MOS sensors,^19^ can thus serve as other markers targetable with the technology. It is envisioned that MOS sensors would also prove useful in the detection of clandestine laboratories, which have a strong smell of ammonia, and could therefore be targetable with sensors.^18,20^

In recent years, there have been wide advancements in the fabrication and performance characteristics of both SnO_2_ (n-type) and Cr_2_O_3_ (p-type) materials. Various nanostructures based on SnO_2_ materials have been developed with low-temperature detection properties. Some of the most recent publications include SnO_2_ porous nanofibers for sub-ppm H_2_S sensing,^21^ SnO/SnO_2_ nanoflowers for formaldehyde detection,^22^ room temperature MoS_2_/SnO_2_ ammonia sensors,^23^ and SnO_2_ inverted opals decorated with Cr_2_O_3_ nanoclusters.^24^ SnO_2_ materials have been combined with zeolites, but mostly as overlayers,^25,26^ instead of mixtures with the actual zeolite. Although n-type materials are generally associated with increased responsiveness to test gases,^27^ great advancements have been made with p-type materials as well. Examples include Pt-Cr_2_O_3_–WO_3_ composite nanofibers for xylene detection,^28^ Cr_2_O_3_ nanoparticle-functionalised WO_3_ nanorods for ethanol detection.^29^ Cr_2_O_3_ thin films have also been explored in combination with Pt-loaded ZSM-5 zeolite films for hydrocarbon detection.^30^

The operating mechanism of MOS sensors is based on a change in the resistance of the sensitive material, which is brought about by changes in the composition of the surrounding atmosphere.^31^ In air and at temperatures in the range of 150–500 °C, oxygen adsorbs on the surface of the MOS material and traps electrons from the bulk, serving to either increase the resistance of the material (n-type semiconductors) or decrease it (p-type semiconductors).^32^ Further details on the operating mechanism of the sensors upon exposure to gases is provided in the discussion. The resistance change is the result of various processes that are thought to occur at the surface, grain boundaries and in the bulk of the sensing material.^33,34^ These processes include adsorption, desorption, redox reactions, catalysis, diffusion, and chemical reactions. These are largely influenced by factors such as the type of sensing material, its microstructure, morphology, concentration of reactive surface sites and of charge carriers, and energetic parameters of adsorption and desorption.^34,35^

This study explores the detection of methyl benzoate with MOS sensors and evaluates whether the modification by zeolite materials significantly enhances the sensor responses of the base materials to this vapour. In addition, Support Vector Machines (SVMs) are used to determine whether or not they can accurately classify different gases of interest. To the best of our knowledge, this is the first time that MOS technology has been used to sample gas in the same way that dogs do to detect cocaine in the field. An initial eleven sensor array based on zeolite-modified SnO_2_ (n-type) and Cr_2_O_3_ (p-type) semiconductor materials is used. SVMs are later used with a four sensor array to classify the data and evaluate whether the array is successful in rapidly and accurately classifying nine different gases of interest. The sensors modified by zeolites enhanced sensor responses dramatically, particularly upon exposure to methyl benzoate, and the selected sensors were 94.1% accurate in classifying all gases just five seconds into gas injection, which is highly promising for the future application of sensors to security scenarios.

## Materials & methods

### Sensor selection rationale

Detailed information on the rationale followed to select the sensors can be found in the ESI.† The initial eleven-sensor array was composed of the following sensors:

#### n-Type

SnO_2_ control, SnO_2_ admixed with 10% (wt) H-ZSM-5, SnO_2_ admixed with 30% (wt) H-ZSM-5, SnO_2_ admixed with 10% (wt) Na-A, and SnO_2_ admixed with 30% (wt) Na-A. After carrying out further work, a sensor based on SnO_2_ admixed with 50% (wt) H-ZSM-5 was also fabricated.

#### p-Type

Cr_2_O_3_ control, Cr_2_O_3_ admixed with 10% (wt) H-ZSM-5, Cr_2_O_3_ admixed with 30% (wt) H-ZSM-5, Cr_2_O_3_ admixed with 40% (wt) H-ZSM-5, and Cr_2_O_3_ overlaid with zeolite H–Y. Details on sensor fabrication can be found in ref. 19.

### Drug-marker detection with MOS gas sensors

**Fig. 1:**
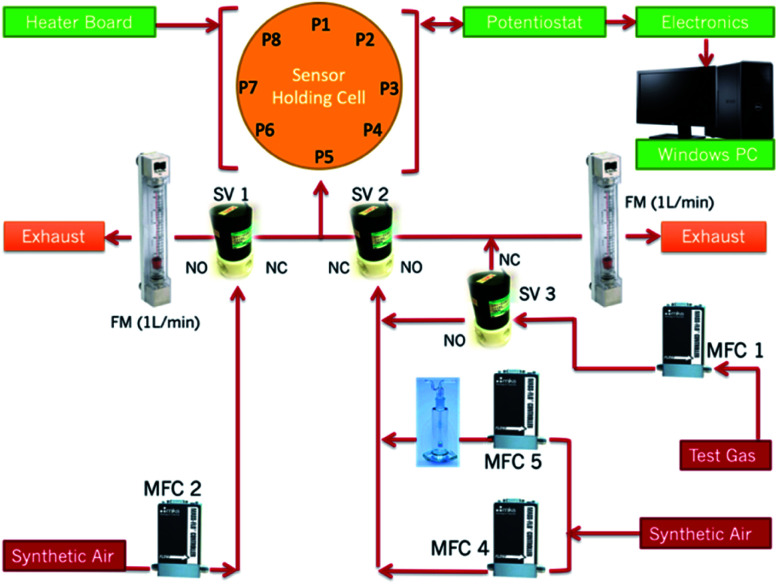
Gas-sensing rig configuration. P1–P8 refer to the port numbers housing the sensors in the sensing chamber. MFC refers to Mass Flow Controllers. SV refers to solenoid valves. FM refers to flow meters. A Dreschel flask was placed after MFC-5 so that tests could be performed under humid conditions and to test liquids for detection.^36^

All tests were performed on a gas sensing rig designed at UCL (Fig. 1). Further details of the rig can be found in ref. 36. Tests were carried out at operating temperatures ranging between 350 °C and 450 °C.

Sensor responses were calculated as follows:

• When using an n-type metal oxide semiconductor material exposed to a reducing gas, the response is *R* = *R*_0_/*R*_g_. When the sensor is exposed to an oxidising gas, the calculation is reversed (*R* = *R*_g_/*R*_0_), where *R* is the responsiveness of the sensor, *R*_0_ is the resistance of the sensor when exposed to air and *R*_g_ is the resistance of the sensor when exposed to a gas.

• When using a p-type metal oxide semiconductor material exposed to a reducing gas, the sensor response is calculated as *R* = *R*_g_/*R*_0_. When the sensor is exposed to an oxidizing gas, the calculation is then *R* = *R*_0_/*R*_g_.

### Test & material details

#### Methyl benzoate

Liquid purchased from Sigma-Aldrich. The liquid was placed in a Dreschel flask and progressively diluted with dry air passing through the flask which was, in turn, connected to the sensor holding cell (Fig. 1). The vapour pressure of methyl benzoate at 20 °C is reported as 0.28 mmHg in the Sigma Aldrich product sheet, which corresponds to a theoretical headspace saturation of 368.4 ppm. In contrast, the vapour concentration of cocaine is reported as 0.25 ppb at room temperature.^37^ With the present configuration of the rig and with the initial source concentration of methyl benzoate, the smallest concentration that could be tested was 37 ppm.

In order to understand whether the sensors were able to discern between the response patterns of methyl benzoate and other gases, they were also independently exposed to other gases of interest. These include: ethanol (100 ppm), ethane (100 ppm), butane (100 ppm), acetone (10 ppm), propane (100 ppm), nitrogen dioxide (1 ppm), ammonia (50 ppm) and toluene (50 ppm) – the source concentration has been indicated in parenthesis (see Table S1†). Multiple concentrations of each of these gases were investigated and later used for SVM analysis. All test gases were obtained and certified by BOC gases.

The reason why these gases were selected is because they are common interferents that could potentially affect the accurate detection of methyl benzoate in the field. This is because they are present in the environment and/or in busy environments, some are used in the manufacture of drugs, and some are abused drugs themselves. Others have been reported to be markers of drugs. Furthermore, although cocaine manufacture commonly occurs in rural settings, urban clandestine labs are being reported more often and as such, it is important to measure environmental vapours as well.^38^

Ethanol is a common interferent that it is present in cosmetics and cleaning products and is also an incredibly common solvent used in drug manufacture.^39^ Acetone occurs naturally in the environment and is also released to the environment as a result of industrial processes, from vehicle exhaust, tobacco smoke, household products, and landfill sites. Ammonia odour has been reported as a common marker of clandestine labs *e.g.* amphetamine and methamphetamine.^38^ Propane and butane are both commonly used in cosmetics, agricultural products, in paints and coatings, and in the manufacture of organic chemicals. Usage of propane cylinders for the manufacture of illegal drugs has also been reported.^40^ Propane and butane are also illegally abused so they could be found in lab environments working towards the manufacture/sale of illegal drugs.^41^ Nitrogen dioxide is a common air pollutant found especially in urban settings. Ethane emissions have risen in recent years in different parts of the world^42^ and it has been reported as an abused drug.^43^ Toluene is an abused drug and is also a common environmental interferent.^44^

Zeolites H-ZSM-5 and H–Y were obtained from Zeolyst International USA (H-ZSM-5 CBV 8014 and Y-zeolite CBV 600) and zeolite Na-A from Advera PQ-Corporation. SnO_2_ was obtained from Sigma-Aldrich and Cr_2_O_3_ from BDH.

### Sensor characterisation

Sensor characterisation was performed on all sensing materials before analysis to ensure that the crystallinity of the material remained unaltered after fabrication.

### X-Ray diffraction (XRD)

XRD was carried out on a Bruker D8 discover diffractometer with Cu Kα1/Kα2 radiation (*λ* = 1.5418 Å) operating at 30 W with a Vantec 500 detector. XRD patterns were collected over the 2*θ* range 15–70°, with a time step of 100 s per step × 3 steps, using a 1 mm collimator.

### Scanning electron microscopy (SEM)

SEM was carried out on a Phillips XL30 environmental scanning electron microscope. The micrographs were collected at a magnification of ×10 000 but additional micrographs were collected at magnifications of ×20, ×1000, ×3000, ×5000, ×20 000, and ×40 000.

### Raman Spectroscopy

Raman Spectroscopy was executed on a Renishaw inVia microscope using a 514 nm excitation laser. Raman spectroscopy was carried out on the powdered materials and the control sensors prior to and after gas and heat exposure to assess any differences caused in the process of testing.

## Results & discussion

### Sensor characterisation

Characterisation results are presented only for the Cr_2_O_3_-based sensors. The characterisation of the SnO_2_-based sensors can be found in ref. 19.

### X-Ray diffraction (XRD)

**Fig. 2:**
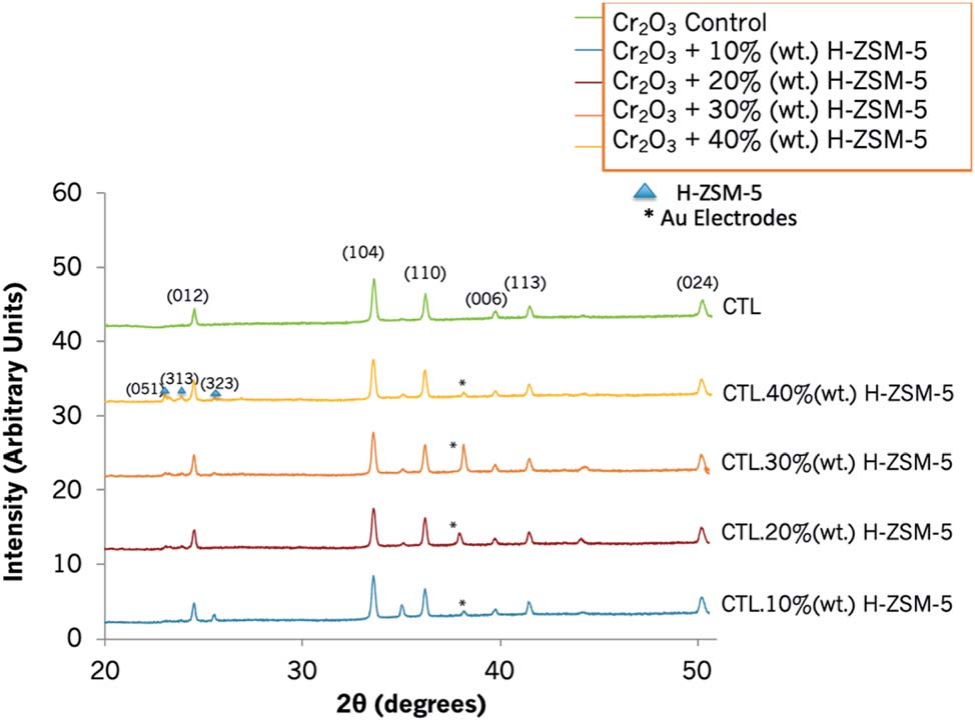
XRD patterns of a control Cr_2_O_3_ sensor and those modified with 10% (wt) zeolite H-ZSM-5, 20% (wt) zeolite H-ZSM-5, 30% (wt) zeolite H-ZSM-5 and 40% (wt) zeolite H-ZSM-5. Peaks have been indexed according to the literature.^47^ Peaks corresponding to H-ZSM-5 have been marked with a triangle and those corresponding to the gold electrodes printed on the alumina substrate have been marked with an asterisk.

The XRD pattern showed Cr_2_O_3_ was hexagonal in structure and had characteristic 2*θ* peaks at 24.4°, 33.6° 36.2°, 39.6°, 41.4°, 50.1° (Fig. 2). The sensors that contained zeolite H-ZSM-5 as an admixture also displayed the characteristic peaks of Cr_2_O_3_ and, as the amount of the zeolite in the sensing system was incremented, a few peaks attributed to it could be appreciated at 2*θ* of 22.9° and 23.9° as seen, for instance, in the sensor containing 40% (wt) zeolite H-ZSM-5.^45^

XRD patterns of Cr_2_O_3_ modified with H–Y overlays are provided in Fig. S2.† The zeolite coating resulted in a higher intensity of the peaks corresponding to H–Y in comparison to Cr_2_O_3_. XRD peaks corresponding to zeolite H–Y were identified at 2*θ* 10.2°, 12.02°, 15.8°, 18.9°, 20.6°, and 23.9°, in accordance with the literature.^46^

### Scanning electron microscopy (SEM)

**Fig. 3:**
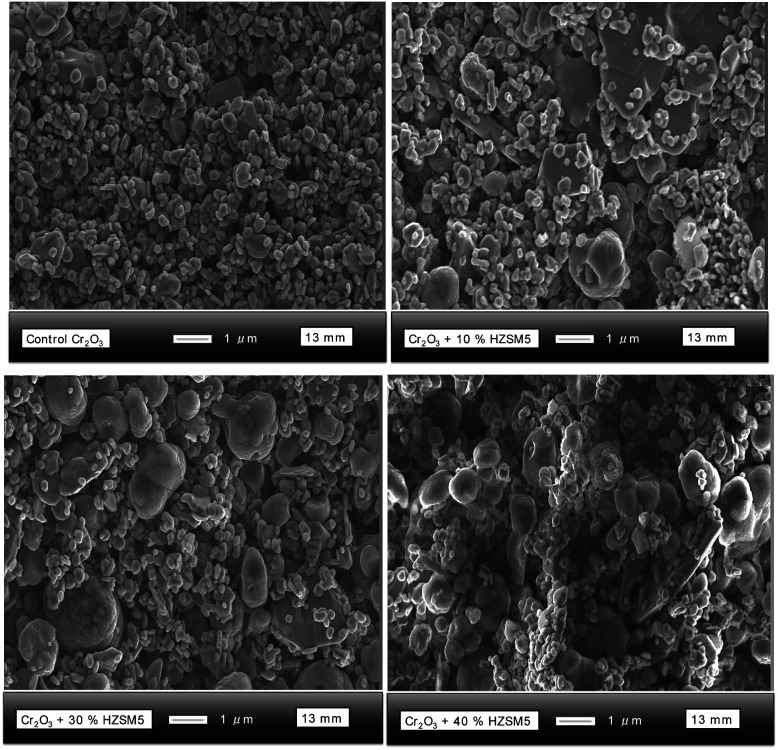
SEM images of Cr_2_O_3_ sensors modified with different amounts of zeolite H-ZSM-5 from 10% (wt) to 40% (wt) H-ZSM-5.

SEM images of the Cr_2_O_3_ sensors modified with different amounts of H-ZSM-5 were taken at a magnification of ×10 000 (Fig. 3). As can be seen in the image corresponding to the control sensor, the particles were loosely stacked together and displayed a porous microstructure with clear voids. The particles exhibited a broad size distribution, ranging from ∼100 nm to ∼400 nm and their shape was not uniform throughout the structure. The progressive increase of zeolite H-ZSM-5 into the sensor structure is clearly visible in the images. The zeolite particles were generally oval in shape and ∼1 μm in size.

The structure continued to appear porous but seemed to become more compact as the amount of zeolite was incremented. Compact or denser films are usually considered to result in poorer sensitivity to test gases in comparison to more porous films.^48,49^ This is because the gas has access, primarily, to the surface of the sensor. There was no evidence of fusion between the oxide and zeolite particles. Nevertheless, the sensor with 10% (wt) H-ZSM-5 displayed slightly different shapes of the zeolite particles, some of which had sharp edges.

Upon closer inspection of the SEM micrographs at higher magnifications (Fig. S3†), there was evidence of some particle agglomeration. As the percentage weight was increased, all zeolite particles showed signs of agglomeration; the particle shapes were oval and they also displayed a rough outer appearance. Cr_2_O_3_ was not easily discernible in the sensor coated with zeolite H–Y (Fig. S4†). Instead, one could observe the zeolite particles of H–Y, which were ∼300–500 nm in size and had a rhomboidal shape. The particles appeared to be interconnected, whereas in the control sensor they appeared loosely packed. At a magnification of ×1 000, the porosity of the zeolite-modified sensor could be easily observed and, although the surface of the control had a much smoother appearance, indicating hindrance to gas diffusion, the zeolite-modified sensor displayed particle agglomerations that protruded from the surface, creating diffusion and interaction pathways for gas molecules.

### Methyl benzoate detection

#### SnO_2_-based sensors: detection of methyl benzoate

**Fig. 4:**
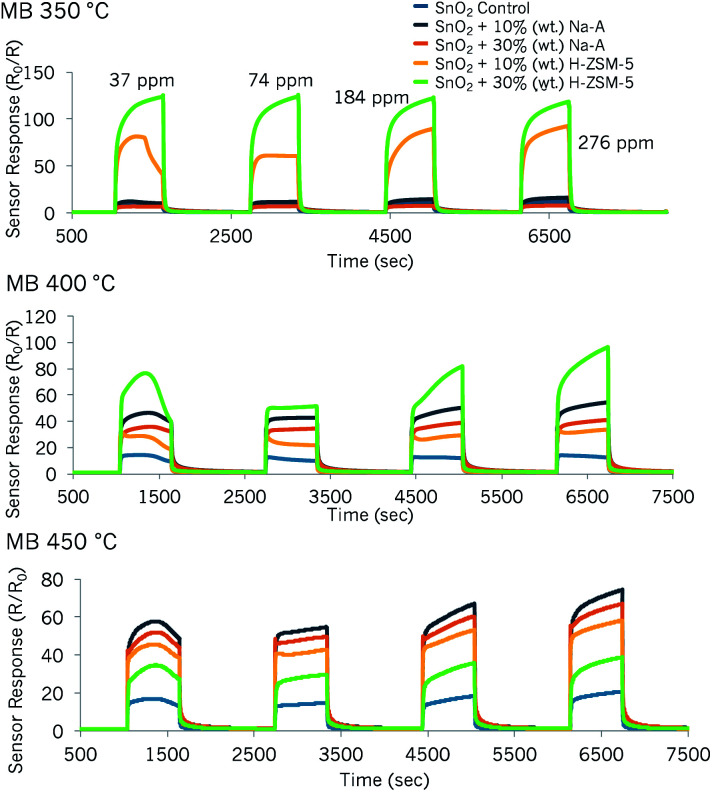
Sensor responses to methyl benzoate (MB) at 350 °C, 400 °C and 450 °C attained with a sensing array based on SnO_2_ zeolite-modified materials by admixture with Na-A and H-ZSM-5.

The results of the SnO_2_ based sensors towards methyl benzoate were very interesting. Including the control SnO_2_ sensor, all sensors proved to be incredibly responsive to methyl benzoate both when supplied with low (*ca.* 37 ppm) and higher (*ca.* 276 ppm) concentrations of the vapour (Fig. 4). Furthermore, excellent response magnitudes were attained at all the operating temperatures. It is noteworthy that the incorporation of zeolite materials was seen to greatly enhance the responses in relation to the unmodified control sensor. Zeolite-modified sensors displayed their highest responsiveness at different operating temperatures. For instance, sensor ‘SnO_2_ + 30% (wt) H-ZSM-5’ was most responsive to methyl benzoate at 350 °C and sensor ‘SnO_2_ + 10% (wt) Na-A’ was most responsive to the vapour at 450 °C (Fig. 4).

Particularly when supplied with *ca.* 37 ppm of the vapour and at operating temperatures of 400 °C and 450 °C, the sensors displayed an unexpected peak shape. This behaviour was also observed with sensor ‘SnO_2_ + 10% (wt) H-ZSM-5’ at 350 °C. The sensors displayed an arch-like shape that had not been observed before with exposure to other test gases.

It was noticed that higher response magnitudes were attained with the first concentration pulse (∼37 ppm) than with the second concentration pulse (∼74 ppm). With the exception of sensor ‘SnO_2_ + 30% (wt) H-ZSM-5’ at 350 °C, all other sensors seemed to reach steady state with the second concentration pulse. The peak shapes changed, once again, with the third and fourth concentration pulses (184 ppm and 276 ppm, respectively), displaying more of a steep increase in sensor response during the gas pulse (exception: SnO_2_ control sensor). Furthermore, while some sensors increased in response with higher concentrations of methyl benzoate, particularly after the second concentration pulse of ∼74 ppm, other sensors' responses were only mildly affected by concentration increments and were saturated *e.g.* ‘SnO_2_ + 30% (wt) H-ZSM-5’ at 400 °C.

The shark-fin shapes seen in some sensor responses indicate slow reaction kinetics in the sensors. The term ‘shark-fin’ has previously been used in the literature to refer to a slow and unsaturated sensor response during the pulse of gas.^50^ This behaviour could be due to the large size of the molecule (kinetic diameter estimated as ≥5.8 Å). As such, it could struggle to diffuse through the microstructure of the sensors. Nevertheless, what was striking was that almost every concentration pulse led to different response shapes. Other studies in the literature have reported odd peak shapes that varied with time and concentration.^1^ These patterns were attributed to multiple gas reactions on the MOS/zeolite system, which led to primary and secondary sensor responses.^1^ It is therefore possible that the results obtained in this study could be indicative of a range of reaction products, the formation of which was strongly dependent on the concentration of methyl benzoate.

At 350 °C, the aforementioned change in peak shape seen as the methyl benzoate concentration was increased was only observed with the sensor containing 10% (wt) zeolite H-ZSM-5. A remarkable increase in sensor response (16-fold enhancement) was obtained with the sensor containing 30% (wt) H-ZSM-5 over that of the control, when supplied with ∼37 ppm methyl benzoate. It must be noted, however, that with this sensor, concentrations exceeding 37 ppm resulted in the same response magnitudes; SnO_2_-based sensors have, in the past, been successful at detecting gases at sub-ppm concentration levels.^16,51^ Because of this, it is thought that SnO_2_ modified with H-ZSM-5 would be a very good sensor candidate to investigate even lower concentrations of this drug marker in future.

SnO_2_ sensors containing zeolite Na-A provided lower responses at 350 °C in relation to the other zeolite-modified sensors. The sensor containing more zeolite in the structure, ‘SnO_2_ + 30% (wt) Na-A’, provided a lower response (*R*_0_/*R*_g_ = 6.5) when supplied with ∼37 ppm of gas than the control SnO_2_ sensor (*R*_0_/*R*_g_ = ∼7.7). The sensor response of ‘SnO_2_ + 10% (wt) Na-A’ was ∼12 at this concentration. The peak shapes on the first concentration pulse were strange in the sense that they saw an increase in resistance during the gas pulse. Furthermore, these two sensors had peak tailing, which was not present in the control sensor. It follows that, at this point, they would be unsuitable for practical applications seeking to detect methyl benzoate at lower operating temperatures *i.e.* 350 °C.

As the operating temperature was raised, the sensor responses of the SnO_2_ sensors modified with zeolite Na-A increased, and provided an improvement in response over the control material. At 450 °C, both sensors modified with zeolite Na-A provided higher responses than those modified with zeolite H-ZSM-5. At 450 °C, the sensors modified by zeolite Na-A only reached steady state with the second concentration pulse (∼74 ppm) and later displayed a similar steep increase in sensor response with higher concentrations of methyl benzoate.

**Fig. 5:**
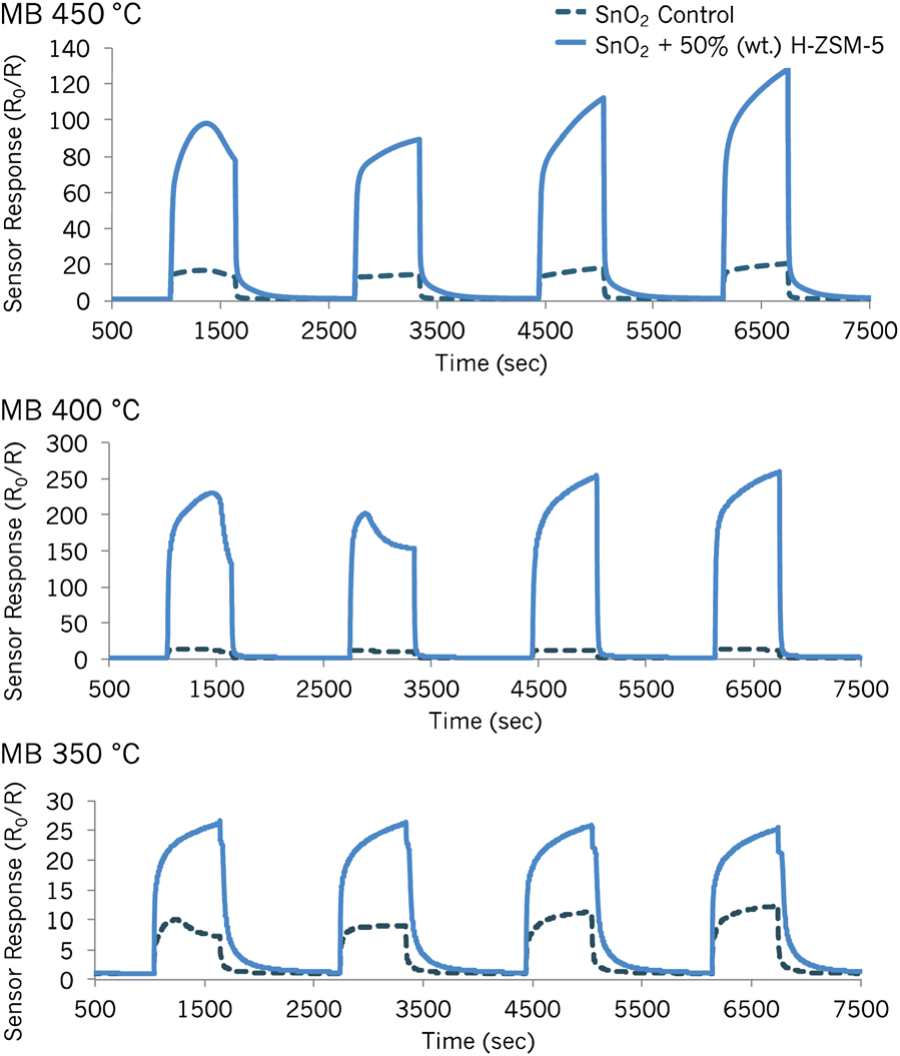
Sensor responses to methyl benzoate (MB) at 350 °C, 400 °C, 450 °C of a control SnO_2_ sensor (dark blue dotted line) and a SnO_2_ sensor modified with 50% (wt) zeolite H-ZSM-5 (light blue line). The concentrations of each gas pulse correspond to *ca.* 37 ppm, 74 ppm, 186 ppm and 276 ppm.

Due to the very interesting results attained with the sensor modified with 30% (wt) zeolite H-ZSM-5 across the temperatures investigated, a new SnO_2_ sensor with 50% (wt) H-ZSM-5 was fabricated and tried for methyl benzoate detection (Fig. 5). As shown in Fig. 5, the SnO_2_ sensor mixed with 50% (wt) H-ZSM-5 provided an outstanding enhancement in sensor response, in relation to the control sensor and other H-ZSM-5 sensors.

The optimal operating temperature to detect methyl benzoate with ‘SnO_2_ + 50% (wt) H-ZSM-5’ was 400 °C as the responsiveness of the sensor was highest. Note that when supplied with ∼37 ppm of methyl benzoate, the zeolite-modified sensor provided a response *R*_0_/*R* = ∼230 at 400 °C and, at higher concentrations such as ∼184 ppm, the response was *R*_0_/*R* = ∼251.

The great enhancements seen in sensor response when incorporating zeolite H-ZSM-5 could be the result of catalytic reactions occurring due to the presence of the zeolite. One plausible explanation for this is that the source molecule is broken down into intermediate products to which the sensing system, as a whole, was very sensitive. Additionally, it is also possible that introducing more zeolite into the sensing framework results in a higher surface area and a more open microstructure that favours the diffusion of methyl benzoate into the sensor bulk. This would allow the methyl benzoate molecules to react with an amplified number of reactive sites as the molecules travel through the sensing layers, promoting an increase in the overall conductivity of the system.^50^ The hydrophobic nature of zeolite H-ZSM-5 may also have an affinity for methyl benzoate (polarity index 0.8),^52^ retaining it or its reaction products well inside its pores.^26^ As can be observed in Fig. 5, the zeolite-modified sensor was able to desorb the molecules successfully when methyl benzoate was no longer supplied to the sensor at 400 °C.

**Fig. 6:**
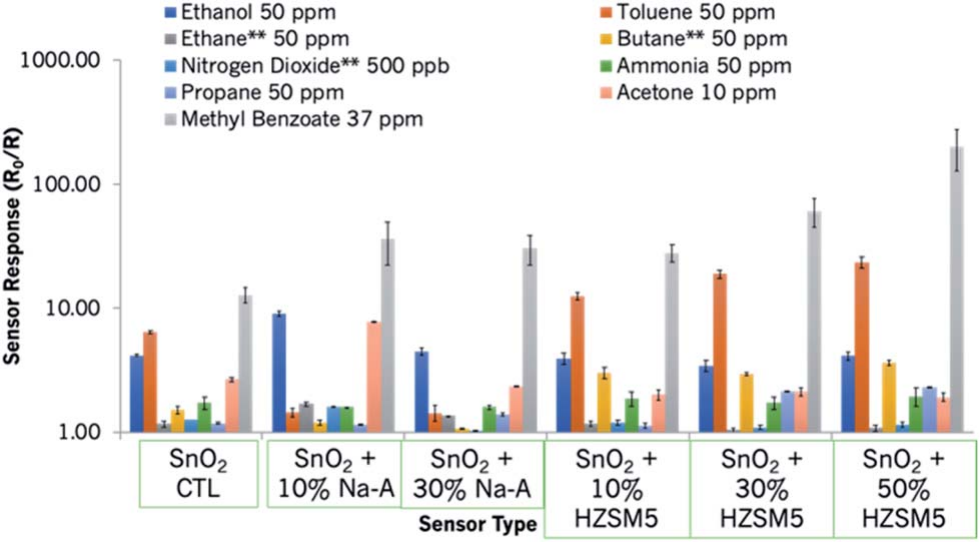
Sensor responses of the SnO_2_-based sensors to a range of gases of interest. The sensors provided resistive responses to those gases marked with an asterisk.

At other operating temperatures, there was evidence of peak tailing, which is unfavourable for practical applications. Nevertheless, commercial products offer temperature cycling steps to clean the surface of the sensors.^2^ A plot illustrating how the sensors behaved upon exposure to a range of gases of interest is shown in Fig. 6. As can be observed in this figure, the zeolite-modified sensors are highly responsive and selective to methyl benzoate (grey bar), in relation to higher concentrations of other gases. A summary of relevant sensor information is presented in Table S2.† In order to get an idea of the applicability of the sensors, the variability of the base material was also tested from one device to another (shown in Fig. S5†), and it was found to be minimal. This is in line with results obtained in other studies that fabricated MOS sensors similarly.^53^

As can be seen in Fig. S6 and S7,† at lower temperatures the variability between tests was more pronounced. It can also be seen that as the concentration of methyl benzoate was raised, the sensor response became saturated. Incorporating zeolites into the sensing system worsened the repeatability in the SnO_2_-based sensors. The degree of variability changed individually for each sensor as the operating temperature was changed. The results provided correspond to an average of the maximum response attained after three repeat tests. Each test (1, 2 and 3) was studied individually to see how the sensors' behaviour varied after each test. When focusing, for instance, on the sensor that was most responsive to methyl benzoate at 350 °C, ‘SnO_2_ + 30% (wt) H-ZSM-5’, test 1 showed that the sensor increased in response as the vapour concentration was raised. In test 2, increasing the concentration of the vapour did not provide different response magnitudes and each pulse reached a response magnitude similar to the last pulse supplied in test 1 (276 ppm). Test 3 also failed to produce enhancements in sensor response with concentration. However, the response magnitude did increase considerably in relation to tests 1 and 2. It is possible that the zeolite was able to retain the test molecule or its reaction products inside its pores or cracks due to the very open microstructure of the SnO_2_-based systems, which did not fully desorb when the vapour supply was switched off. As further tests were carried out, it is possible that the molecules retained inside the structure continued to react with reactive sites and were subsequently able to penetrate deeper into the sensing layer of the material, eventually resulting in higher sensor responses than those attained in test 1. Because the sensor did not reach steady state during the duration of the gas pulse this suggests that there were enough reactive sites available for the molecules to interact with ref. 54.

It must be noted that the heterogeneity of the microstructure in the sensors admixed with zeolite could also lead to the observed variability among tests; the gas interacts differently with different areas of the system and charge transfer may have been affected by this.^55^ Better insight into what might be occurring could potentially be reflected in the sensor resistance change from test to test; from test 1 to 3, the sensor resistance increased progressively. If MB failed to desorb off the sensor effectively once the vapour was turned off, it is possible that when oxygen was reintroduced to the sensor it was able to interact with remaining molecules inside the sensor, abstracting more electrons from the sensor and thus increasing the baseline sensor resistance.

#### Cr_2_O_3_-based sensors: detection of methyl benzoate

**Fig. 7:**
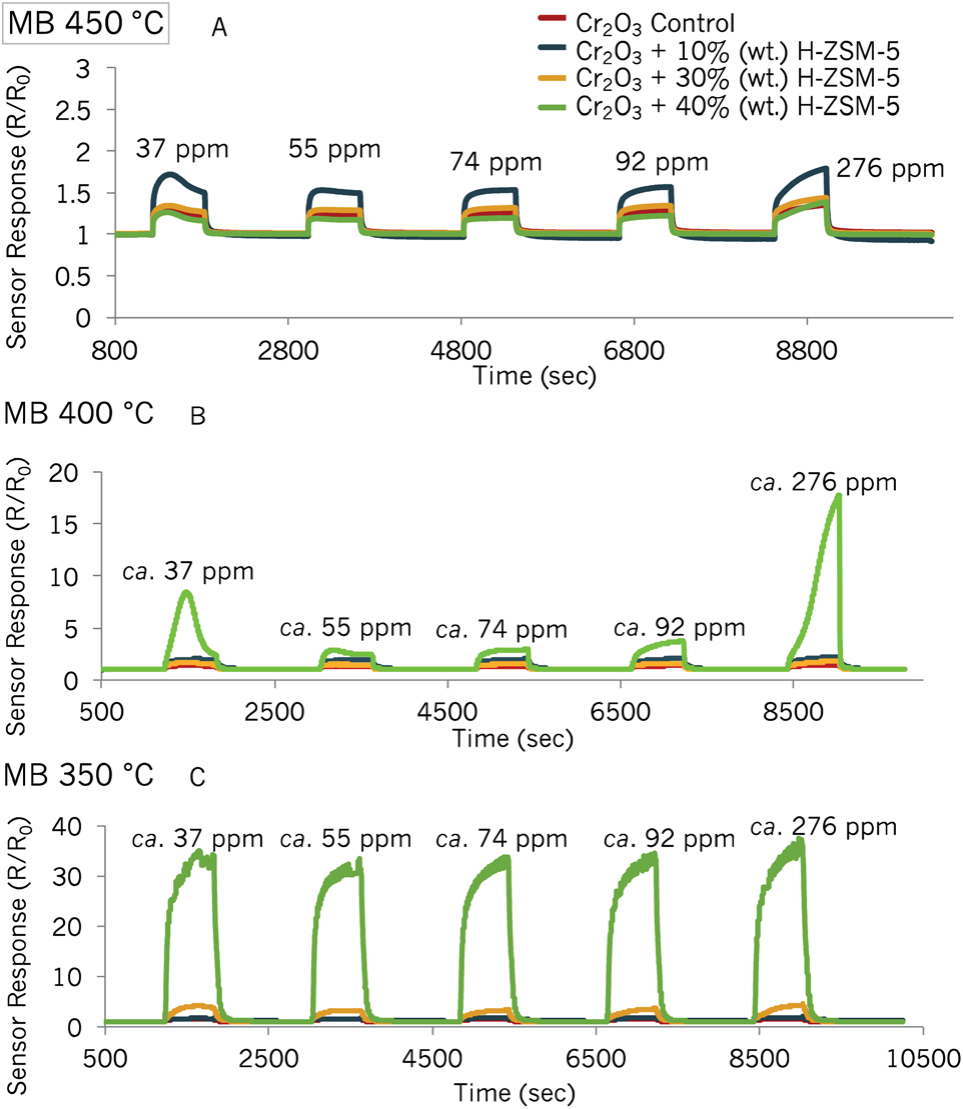
Sensor responses of Cr_2_O_3_ control sensor and Cr_2_O_3_ modified by 10% (wt), 30% (wt) and 40% (wt) zeolite H-ZSM-5 to MB at 450 °C (A), 400 °C (B) and 350 °C (C).

Similar tests were carried out with Cr_2_O_3_-based sensors, modified by mixing zeolite H-ZSM-5 with the base material. The sensor responses of p-type semiconductor materials is commonly reported to provide more conservative responses than n-type materials.^36^ While the sensor responses of the Cr_2_O_3_ materials were lower, they were still particularly high in some of the sensors fabricated. Sensor exposure to methyl benzoate is shown in Fig. 7A–C. As seen in the graphs, the Cr_2_O_3_ zeolite-modified sensor that was most responsive to methyl benzoate was ‘Cr_2_O_3_ + 40% (wt) H-ZSM-5’ both at 350 °C and 400 °C. At an operating temperature of 450 °C, sensor responses were all rather similar and *R*/*R*_0_ < 2 for all sensors. The magnitude of response of ‘Cr_2_O_3_ + 40% (wt) H-ZSM-5’ was practically unaffected by methyl benzoate concentration increments. Furthermore, the latter sensor failed to reach steady state during the gas pulse. At 400 °C, the same sensor seemed poorly responsive to the vapour until it was supplied with 276 ppm, and saw a 12-fold increase in sensor response in relation to the control sensor.

The odd peak shape that was observed with the first concentration pulse in the SnO_2_-based sensors was also observed in some of the Cr_2_O_3_-based sensors at 450 °C and 400 °C. The first concentration pulse also resulted in higher magnitudes of sensor response than the second concentration pulse that followed in some sensors.

Response *vs.* concentration plots are shown in Fig. S8 and S9.† The tests at 350 °C were carried out twice and the results in Fig. S8† show little variability in sensor responses between tests. As can be seen in these two figures, the response magnitudes towards methyl benzoate were, indeed, smaller than with the SnO_2_-based sensors. However, the variability between repeat tests was also reduced. The sensor most attractive for the detection of trace concentrations of methyl benzoate would be that containing 40% (wt) H-ZSM-5 and at an operating temperature of 350 °C, since it was much more responsive at this temperature and the variability was low, with the exception of the first methyl benzoate pulse.

Fig. S10 and S11† provide an example of how differently the n-type and p-type sensors responded to methyl benzoate at two different temperatures and how sensor variability among repeat tests could be a key determinant factor in selecting which sensors would be good candidates for further testing.

As observed in a different paper by the authors,^19^ variability was generally reduced when lower vapour concentrations were supplied to the sensors. Given that in practical settings methyl benzoate detection would be needed at much lower concentrations, it is expected that sensor variability would be less of an issue.

**Fig. 8:**
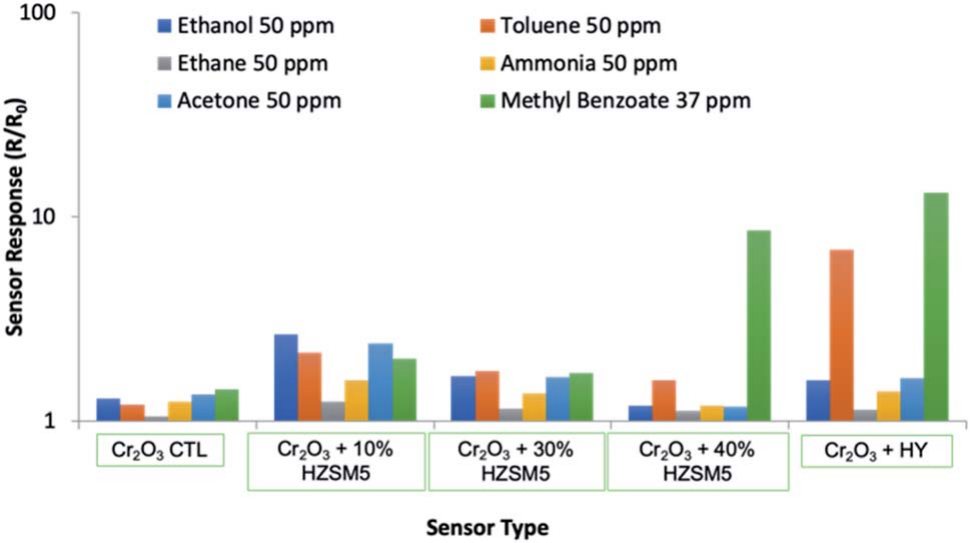
Sensor responses of the Cr_2_O_3_-based sensors to a range of gases of interest.

Fig. 8 shows the Cr_2_O_3_-modified sensor responses to a range of gases of interest. As can be observed, sensors modified with 40% (wt) zeolite and with layers of zeolite H–Y were selective towards methyl benzoate, in relation to other gases that were supplied at higher concentrations. It can be seen that the H–Y sensor was also responsive to toluene, which is due to the zeolite adsorbing toluene well in its super-cages.^56^ These two sensors are therefore very good candidates for the selective detection of methyl benzoate. One of the concentrations tested, 276 ppm, was used as an example to investigate the sensors' response and recovery times at 350 °C and 400 °C in n-type and p-type systems.

Results are shown in Table S2 in the ESI.† The results are based on the last repeat test performed. In general, the incorporation of zeolites in n-type systems led to either faster or comparable sensor response times when compared to those obtained with the control sensor and they served to increase the recovery times in relation to the control SnO_2_ sensor. Conversely, zeolite incorporation led to comparable or longer response times in zeolite-modified p-type systems in relation to the control Cr_2_O_3_ sensor and the recovery times were considerably improved over those seen with the control, particularly with higher zeolite loadings in the sensing system. More specifically, the SnO_2_ based sensors generally responded and recovered more quickly (in ∼2 seconds) at 400 °C than at 350 °C, as expected.

At 350 °C, the sensors modified with zeolite H-ZSM-5 responded slightly more quickly than the control SnO_2_ sensor or those containing zeolite Na-A. Furthermore, at this temperature, the response times were slightly longer as the zeolite loading increased. At 400 °C, the sensor recovery times increased with higher loading of zeolites in the sensing system.

Sensors containing zeolite Na-A had longer recovery times than those containing zeolite H-ZSM-5. The latter can potentially be the result of zeolite H-ZSM-5 catalysing reactions and breaking the methyl benzoate molecule down to products whose diffusion through the system was more straightforward. Zeolite Na-A is not known as a cracking agent and it is also possible that the methyl benzoate molecule or its reaction products experienced more difficulty in desorbing off a sensor microstructure that was perhaps more compact in nature.

In an attempt to identify the presence and nature of by-products, sensors were connected to a mass spectrometer to try and measure – in real time – if there were any detectable by-products being formed during the gas pulse at different sensor temperatures. Unfortunately, with this method, we were unable to detect other products other than the gas supplied. This was potentially due to the very low concentration of the by-products formed. ATR-FTIR was also tested on the surface of the sensor straight after gas exposure but the sensors crumbled upon contact with the probe due to poor mechanical robustness and analysis was thus not possible. Nevertheless, physicochemical characterisation techniques were carried out before and after gas sensing, and no differences were found before and after analysis (Fig. S13†).

In comparison to the n-type systems, the Cr_2_O_3_-based sensors took a much longer time to respond and less time to recover. The zeolite-modified sensors took longer times to respond than the control at 400 °C but *τ*_90_ was very similar to the control sensor at 350 °C.

It seemed to be the case that with p-type systems, having more zeolite in the structure assisted in vapour desorption both at 400 °C and at 350 °C. The Cr_2_O_3_ sensor with 10% (wt) H-ZSM-5 responded very similarly to the control sensor (∼360 seconds at 400 °C and ∼343 seconds at 350 °C) but recovered much more slowly at both temperatures. Although it was expected that at higher temperatures the sensors would respond more quickly due to the thermal energy accelerating the rate of the reaction processes, it was found that they were comparable to, but longer than, the 350 °C response times. It must be noted that the Cr_2_O_3_ sensor that contained 40% (wt) H-ZSM-5 exhibited a great increase in sensor response at 276 ppm (Fig. 7B), in relation to lower concentrations and it displayed an odd peak shape at 400 °C. This could be indicative of different reaction products progressively interacting with the sensor as the vapour was being supplied, which could have led to the much longer response times seen in this sensor at this temperature. The performance of some Cr_2_O_3_-based sensors was monitored for almost a month to understand how exposure to other gases and sensor cycling could affect the sensors over time (see Fig. S12†). As shown in this figure, the variation between sensor responses was found to be negligible.

It is possible that certain sensor microstructures, which appeared to be more open in nature, could favour the diffusion of larger molecules and enable the subsequent reaction of the molecules in the inner layers of the bulk.^57^ In turn, some reaction products may be retained as a result of higher affinity with the system or due to their size and/or shape and may continue to interact with the zeolite particles and the pores within.^57^ Issues of long response and recovery times have been noted with toluene, another aromatic molecule of larger size.^36^

It is thought that the n-type systems responded more quickly and the conductivity of the system was more prominent upon exposure to gases because the microstructure was more porous and the particle size of the base material was much smaller and provided a higher surface area for gas interaction than the p-type systems.

If this was true, and some of the sensing microstructures used here limited the interaction between the gas and the sensing system to only the outer layers of the sensor due to their compactness, they could offer less variability between tests. The longer response times in such a case would be due to the poor efficiency of the molecule in passing through the cavities in the system and the shorter recovery times due to a shorter desorption pathway.

It is thought that with Cr_2_O_3_ sensor exposure to methyl benzoate, the system containing more zeolite is able to catalyse reactions more efficiently than those that have less zeolite. This, in turn, enables the diffusion of smaller molecules into the system, as suggested in other studies that carried out GC-MS to investigate the reaction products on zeolite-modified sensors.^58^ p-Type systems have been reported to be less responsive to flammable gases than n-type systems. The more compact microstructure of the Cr_2_O_3_ based sensors may limit the access of the molecules further down inside the bulk, leading to smaller response magnitudes but, also, to fewer issues with repeatability.

#### Exposure to methyl benzoate of the Cr_2_O_3_ sensor modified by overlayers of zeolite H–Y

**Fig. 9:**
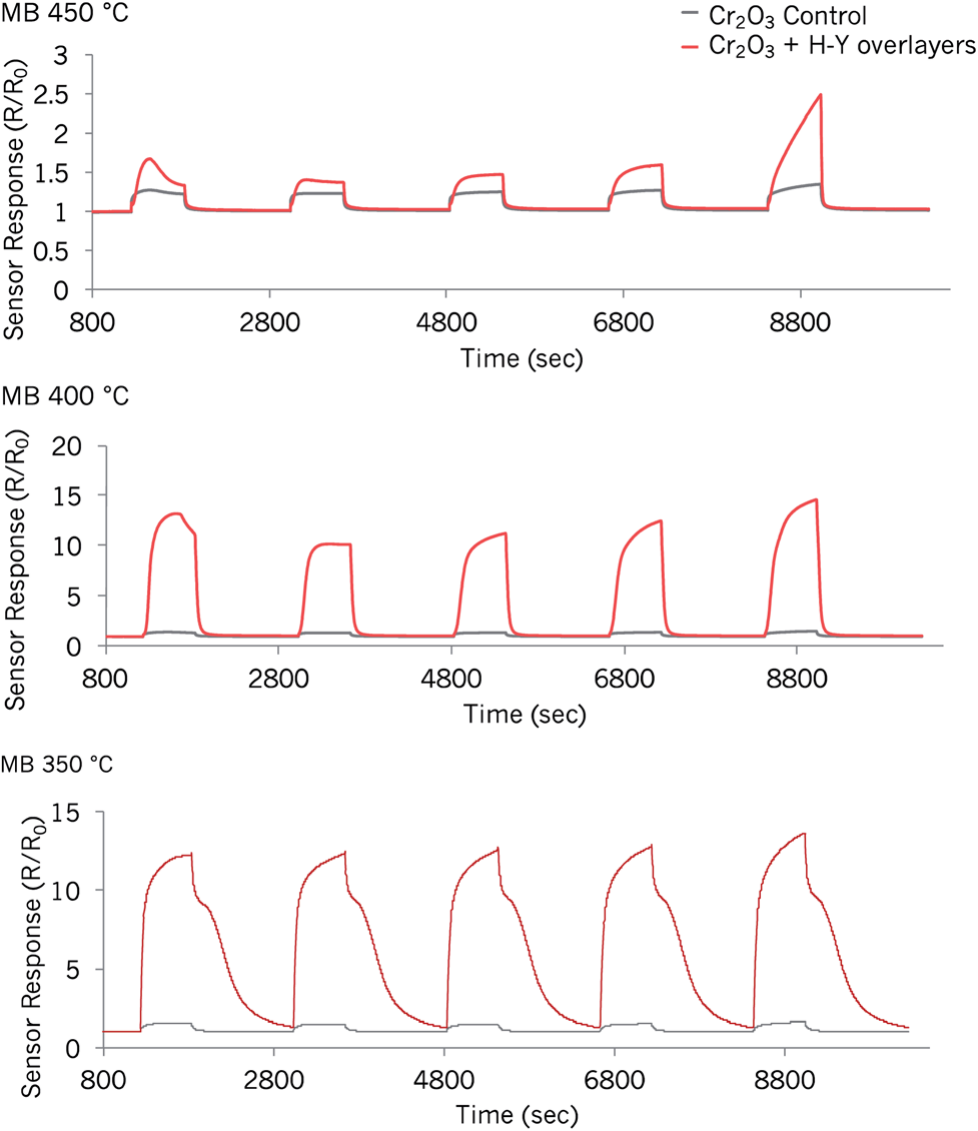
Sensor responses to methyl benzoate of a control Cr_2_O_3_ sensor and one modified by coatings of zeolite H–Y at three different temperatures. The concentrations tested correspond to those in Fig. 6 and 5, 37 ppm, 55 ppm, 74 ppm, 94 ppm and 276 ppm.

In a previous study, Cr_2_O_3_ modified with overlayers of zeolite H–Y was found to provide great improvements in sensor responses to toluene.^36^ Due to the larger kinetic diameter of methyl benzoate, the potential of the sensor for its detection was also evaluated, with the expectation of observing high responsiveness to the vapour as well. The results of this are shown in Fig. 9 below.

The best operating temperature of the zeolite-modified sensor for the detection of methyl benzoate was 400 °C: it provided the highest response magnitudes to the supplied vapour concentrations and the sensors recovered faster than in the test carried out at 350 °C. The first concentration pulse led to higher response magnitudes than the pulse that followed. When supplied with *ca.* 37 ppm of methyl benzoate, the zeolite-modified sensor provided a ∼9-fold increase in sensor response over the control sensor at 400 °C. Response *vs.* concentration plots also showed that the response became saturated with concentration increments.

The sensor response and recovery times were also investigated in this system (Table S2†), using the 276 ppm methyl benzoate concentration as an example of how the sensors performed. As observed with other Cr_2_O_3_-modified sensors, the response times (*τ*_90_) were longer than the recovery times. Nevertheless, the response times of the zeolite-modified sensor were shorter in relation to those seen in the control sensor and in previously-reported Cr_2_O_3_ zeolite-modified systems at 400 °C. However, they were still considered to be too long for practical applications. Having thinner printed layers of the base material could perhaps circumvent this issue.^59^ The lower response magnitudes that may be attained in such a case, could be addressed by reducing the particle size of the base material, for instance, and/or by doping with noble metal catalysts.^25^

### Sensing mechanism

In terms of the operating mechanism of both n- and p-type materials, when an n-type semiconductor is exposed to air, atmospheric oxygen will populate the surface of the material. At low temperatures, oxygen may adsorb on the surface in molecular (O_2_) or atomic form (O).^60^ The high electronegativity of oxygen atoms and molecules allows them to act as electron acceptors, thus extracting and trapping electrons from the bulk of the sensing material, becoming ionised to form O^−^, O_2_^−^, O^2−^ species.^60,61^ The type of oxygen species that dominate on the surface depends on the operating temperature and humidity conditions.^62^ The extraction of electrons from the sensing material results in the creation of additional surface states within the band gap of the material.^61^ In turn, trapped electrons form an electron depletion layer near the surface of the material, termed EDL, which creates a potential barrier (or Schottky barrier) between adjoining grains.^27^ This establishes the baseline resistance of the sensor in air. Thus, supplying a reducing gas *e.g.* carbon monoxide (CO) to an n-type semiconductor *e.g.* SnO_2_ leads to its reaction with chemisorbed oxygen species at the surface, producing carbon dioxide (CO_2_), which then desorbs off the surface.^32^ The occurrence of this reaction leads to a change in the concentration of oxygen species at the surface of the material. Oxygen then oxidises the gas and the trapped electrons are injected back into the semiconductor.^27,63^ This process reduces the thickness of the EDL, lowering the height of the potential barrier between adjoining grains, and leading to an increase in the conductivity of the sensing material.^64^ Conversely, when an n-type material is exposed to an oxidising gas such as NO_2_, the gas extracts electrons from the metal oxide material, reducing the concentration of the majority carriers in the material. This leads to an increase in the size of the EDL and the resistance of the sensor material increases. p-Type semiconductors such as Cr_2_O_3_ behave oppositely. When molecular oxygen adsorbs on the surface of a p-type semiconductor, oxygen will ionise, extracting electrons from the sensing material.^65^ This leads to the formation of a hole accumulation layer, (HAL), at the surface of the material.^27^ As a consequence, the oxidation reaction between carbon monoxide and oxygen species results in a reduction of the concentration of the majority charge carriers – holes – as the electrons are injected back into the oxide and recombine with the holes in the valence band. This leads to an increase in the sensor resistance with exposure to a reducing gas. The opposite trend is seen when a p-type material is exposed to an oxidising gas. The oxidising gas can remove electrons from the MOS, increasing the concentration of holes and therefore the conductivity of the material.^65^

Zeolites have been used in combination with gas sensors with a number of positive effects over the years. Bearing in mind that the response of a sensor to a gas can be affected by different variables, such as the diffusion of a gas through a material, the type of reaction(s) that occur on the surface of the sensor, and the reaction kinetics, the incorporation of zeolite materials to the sensor structure is likely to have an important effect on these.^66^

The diffusion of the gas through a sensor that contains a zeolite will lead to the diffusion of the gas through its framework. This, in turn, slows down the response rate of sensors. The topology of zeolites has previously been used strategically to induce discrimination among gases of similar structure – when used as a membrane, zeolite-modified gas sensors act as sieves and enable the passage of molecules through the system according to size and shape. In addition, zeolites make the whole sensing system a much more open microstructure that has high porosity and is of large surface area. This means that zeolites introduce a higher proportion of reactive sites for the gas to interact which contributes to the increased conductivity of the system. Zeolites are also known to operate as transformation elements. Previous research has shown that zeolite incorporation into sensing systems can lead to hydrocarbon cracking and to the production of a number of products to which the sensing element may be more or less responsive.^58^ When the sensor response to a gas is seen to increase, it is because of higher sensitivity to the reaction products.

The sensors reported in this study have been exposed to a number of gases of interest in addition to methyl benzoate. It is thought that it is a combination of the variables discussed above that led to the enhanced responses observed in this study. For instance, the porous microstructure of the zeolites and the reactions taking place in the zeolite framework led to the significantly enhanced sensor responses observed. The incorporation of zeolites was seen to generate enhanced responses to most gases, as seen in Fig. 6 and 8. However, zeolite-based sensors are particularly sensitive and selective to methyl benzoate. It is thought that the sensitivity towards methyl benzoate of sensors containing zeolite H-ZSM-5 is also related to the hydrophobicity of the zeolite and the low polarity of the molecule. In fact, other molecules of lower polarity, such as toluene (polarity index 2.4),^67^ also showed an affinity towards sensors containing this zeolite. In contrast, sensors containing a more hydrophilic sensor, such as Na-A, although they too were responsive to methyl benzoate, they were also responsive to polar molecules such as ethanol and acetone and not to toluene.

The next section looks at the classification accuracy of nine different gases using only four out of the eleven gas sensors utilized in this study. SVMs results have focused on data obtained at 400 °C.

### Support vector machines

Four sensors based on n-type (SnO_2_) and p-type (Cr_2_O_3_) MOS were selected to test whether a WEKA classification algorithm could build a model able to discriminate accurately between a range of gases, including methyl benzoate.

Due to practicality concerns, it was decided that instead of testing all eleven sensors with the SVM, only four would be chosen for analysis because it is a much more reasonable number of sensors for integration into a portable device. The sensors used were: ‘SnO_2_ + 10% (wt) Na-A’, ‘SnO_2_ + 10% (wt) H-ZSM-5’, ‘Cr_2_O_3_ + 40% (wt) H-ZSM-5’, ‘Cr_2_O_3_ + HY’. These two Cr_2_O_3_-based sensors were chosen due to the high responsiveness of the sensors towards MB and the contrasting responses obtained upon exposure to other gases. The other Cr_2_O_3_-based sensors were not incorporated into the final array because the responses provided to other gases were quite similar and it was thus anticipated that this would not help with gas discrimination. These specific SnO_2_ sensors were chosen because although they both provided a high response towards methyl benzoate, they also provided very contrasting responses to other gases of interest and/or they provided faster response and recovery times than other sensors, which would be useful for practical applications. Further information about the SVMs used can be found in the ESI.†

A Sequential Minimal Optimisation (SMO) SVM was used as a classifier. The SMO approach used to train the SVM solves multi-class problems using a one-*versus*-one strategy. The cost function ‘*C*’ was subsequently modified to find the optimal parameters for classification (refer to ESI† for further details on the technique). There are several model selection methods that assist in minimising the expectation of errors when the classifiers are used as diagnostic tools. An example of this is the leave-one-out approach, which removes a sample from the training set and builds the decision function to infer the class type the removed sample belongs to. This approach was employed as a tool to evaluate the generalisation performance of the classifier. The classifier produces a confusion matrix (Table 1), which tells you how many times a gas is accurately classified as its true label and how many times it is confused with a different gas.

**Table 1:** Confusion matrix provided by the Weka software, using an SMO algorithm and a Polykernel function to build the model with a cost function of *C* = 200, providing a 94.1% accuracy in correctly classifying the data according to gas type with the following four sensor array: ‘SnO_2_ + 10% (wt) Na-A’, ‘SnO_2_ + 10% (wt) H-ZSM-5’, ‘Cr_2_O_3_ + 40% (wt) H-ZSM-5’, ‘Cr_2_O_3_ + H–Y’

Classified as →	A	B	C	D	E	F	G	H	I
A = ethane	5	0	0	0	0	0	0	0	0
B = ethanol	0	5	0	0	0	0	0	0	0
C = nitrogen dioxide	0	0	5	0	0	0	0	0	0
D = ammonia	0	0	0	5	0	0	0	0	0
E = methyl benzoate	0	0	0	0	5	0	0	0	1
F = acetone	0	0	0	0	0	9	0	1	0
G = butane	0	0	0	0	0	0	4	1	0
H = propane	0	0	0	0	0	0	0	5	0
I = toluene	0	0	0	0	0	0	0	0	5

The classification accuracy of the SVM classifier using the polykernel function was 94.1% when using *C* = 200 (Table 1). The data used to build the model included data points of the entire sensor transients; the data inputted was at 5, 10, 50, 100, 200, 300, 400, 500, and 600 seconds after the gas injection. Other important data points included sensor responses at different gas concentrations. 5/6 times methyl benzoate was accurately classified, which is very encouraging. Ammonia, a commonly targeted gas in security applications, was accurately classified 100% of the time. Gases such as ethanol, ethane, and toluene, which are also abused drugs (and also markers needed in medical applications to identify diseases such as lung cancer and alcoholism) were all discriminated successfully. Acetone, a common marker for diabetes, was accurately classified 9/10 times. Other gases relevant to environmental and air-quality monitoring were also accurately classified *e.g.* ammonia, nitrogen dioxide, ethane and toluene. Ethanol, a common interfering gas, was also classified correctly.

It was also interesting to understand whether the SVM classifier would be able to accurately classify the gases after 5 seconds into the gas injection, instead of after 600 seconds. After 5 seconds into the gas pulse, the model was also 94.1% accurate in correctly classifying the labels according to the type of gas (these results were attained with the polykernel function and *C* = 200). The polykernel function generally appeared to perform very well. However, in order to substantiate this finding, an alternative classification algorithm based on a random forest was also used. It was found that the classification performance of the random forest was 70.6%, a scale of difference that suggests a need for further investigation in advance of deploying this practically. Nevertheless, the results thus far are promising: accurate classification was obtained very quickly (in the order of seconds) and in a very inexpensive manner. Ultimately, this methodology could provide users with rapid triage in future: in large-scale operations it could provide an automated initial indication of whether there was a need for further investigation. For this reason, these results are encouraging: they support the need for further research with zeolite-modified sensors for security applications. More specifically, they highlight the potential for advancing illicit drug marker detection though a combination of sensor design and machine learning. Future work will attempt methyl benzoate detection in the presence of interferences and will also involve the investigation of much lower concentrations of the vapours tested.

## Conclusions

The results in this study have shown that targeting vapours emanating from liquid samples may lead to great enhancements in sensor responses. The results are promising because many abused drugs are liquids, and illicit drugs can be concealed in solvents as a trafficking strategy.

Although it is still early days, it is considered that being able to detect methyl benzoate, a by-product of cocaine, and having attained such promising enhancements in zeolite-modified sensor responses when compared to the control sensors, SnO_2_ and Cr_2_O_3_, is very encouraging. As such, this line of work should be pursued further in future. Although zeolite incorporation introduced huge improvements in sensor responses, it is acknowledged that they may also introduce great variability in sensor responses between repeat tests, which would be problematic in real life applications. Attaining a more homogenous microstructure and reducing the particle sizes might improve results further. It would be very interesting to incorporate metal catalysts to n-type and p-type materials to understand if the variability in sensor response would be minimised when compared to zeolite-modified sensors and whether repeatability could also be improved at lower operating temperatures.

Classification tools were employed to understand whether different classifiers could be used to build models that would fit the input data, such that they could classify it correctly into gas type. Using an SVM classifier, an accuracy of 94.1% was attained in classifying the data according to gas type with only four sensors and just five seconds into the gas injection. Other classifiers *e.g.* random forests provided lower classification accuracies (70.6%). Although, at present, it is difficult to know whether methyl benzoate could be detected in more complex environments and how the sensors would respond to even lower concentrations of the vapour, these results are positive. The results presented here reinforce the potential of this line of research for a wide range of purposes, including environmental, air-quality, medical and security-based applications.

## Conflicts of interest

There are no conflicts to declare.

## Supplementary Material

RA-010-D0RA03687K-s001
