# Supplementary material for: Cocaine by-product detection with metal oxide semiconductor sensor arrays
Source: RSC Adv. 2020 Aug 4;10(47):28464–77. doi: 10.1039/d0ra03687k (PMC9055647; doi:10.1039/d0ra03687k)
Supplement: RA-010-D0RA03687K-s001 [file RA-010-D0RA03687K-s001.pdf]

Electronic Supplementary Material (ESI) for RSC Advances.
This journal is © The Royal Society of Chemistry 2020

Supplementary Information

Cocaine by-product detection with metal oxide semiconductor sensor arrays.

Paula Tarttelin Hernández[a]†*, Stephen Hailes[b], Ivan P. Parkin[a]

[a] Department of Chemistry, 20 Gordon St, University College London, London WC1H 0AJ

[b] Department of Computer Science, 66-72 Gower Street, University College of London, London WC1E 6BT

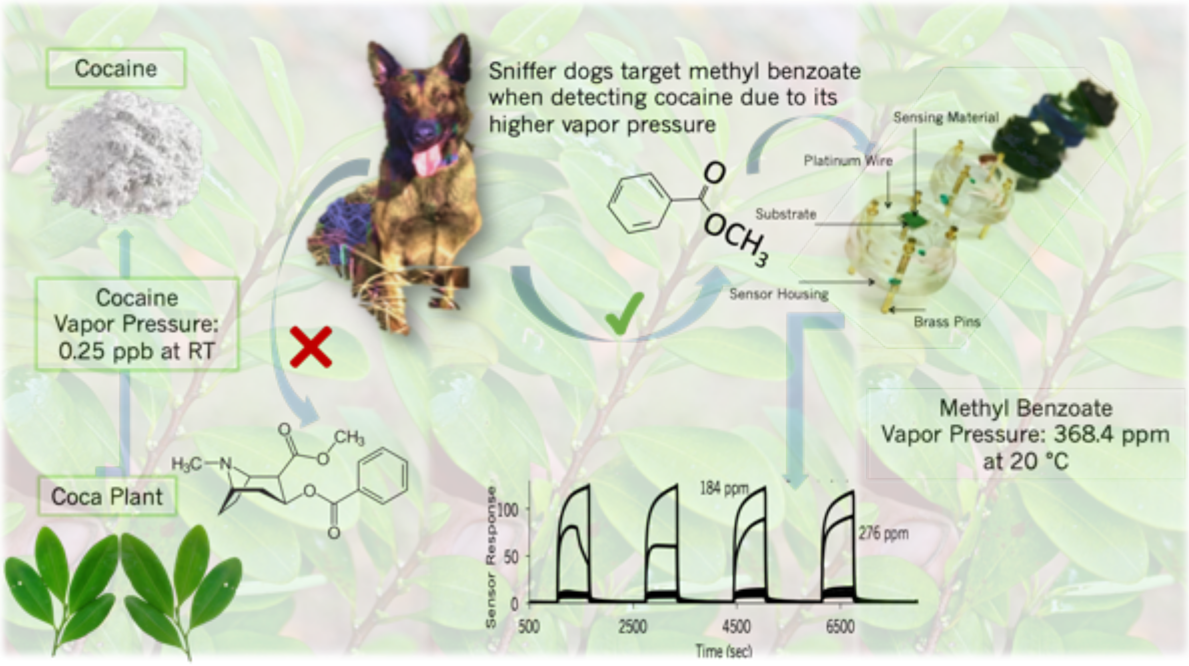

Figure S1. Detection dogs target methyl benzoate when looking for cocaine as it is an odorous by-product of the illicit drug. In this study, it was investigated whether MOS sensors could also successfully detect the drug by-product.

XRD patterns of Cr_2O_3 modified with overlays of zeolite H-Y

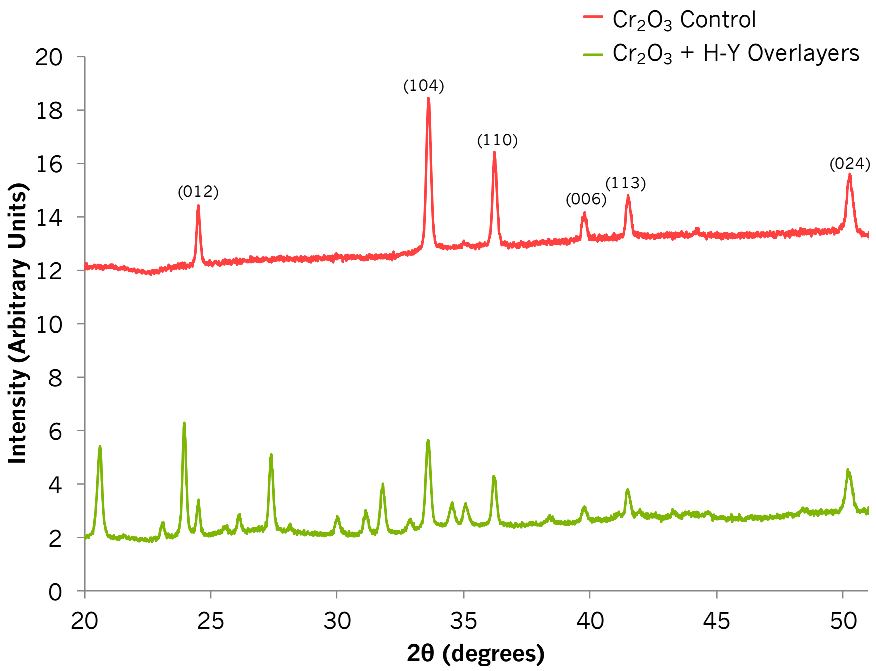

Figure S2. XRD patterns of a control Cr_2O_3 sensor and a Cr_2O_3 sensor modified by screen-printed layers of zeolite H-Y on top of it. Peaks have been indexed according to the literature.(1)

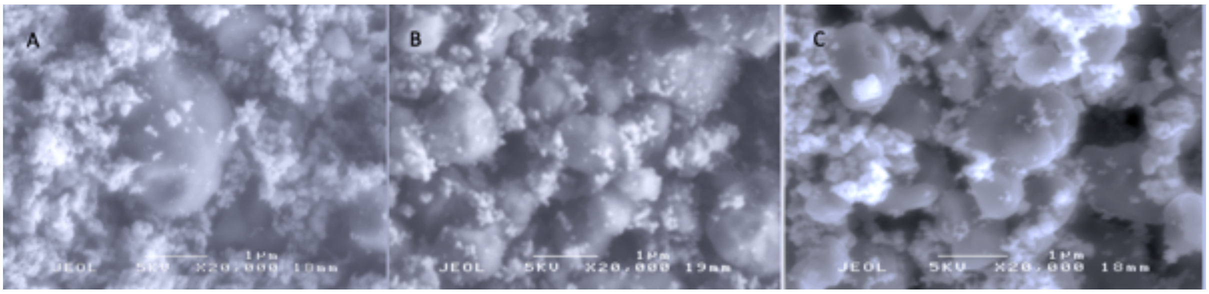

Figure S3. SEM images of A) SnO_2 admixed with 10% (wt.) H-ZSM-5, B) SnO_2 admixed with 30% (wt.) H-ZSM-5, and C) SnO_2 admixed with 50% (wt.) H-ZSM-5.

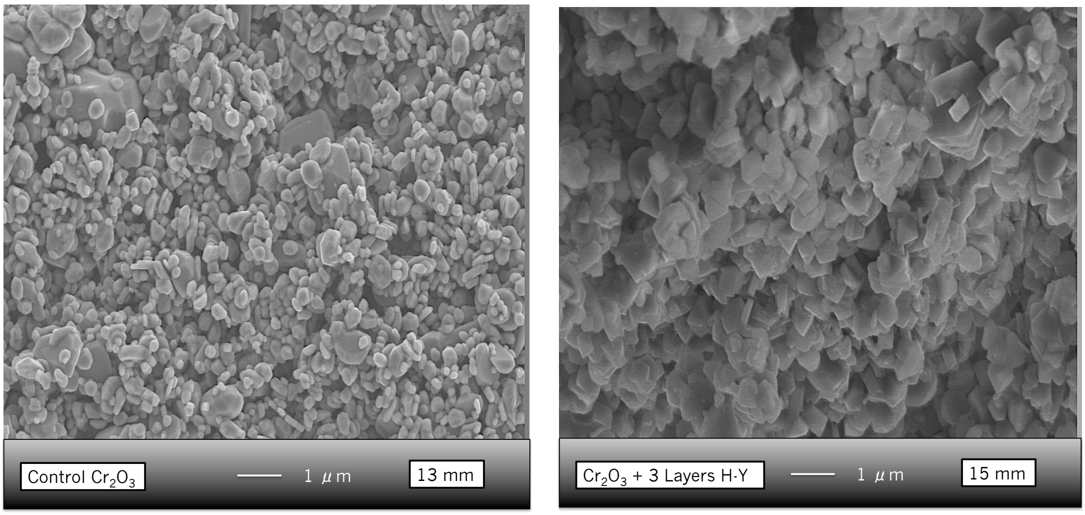

Figure S4. SEM images of a control Cr_2O_3 sensor (left) and a Cr_2O_3 sensor coated with three layers of zeolite H-Y (right).

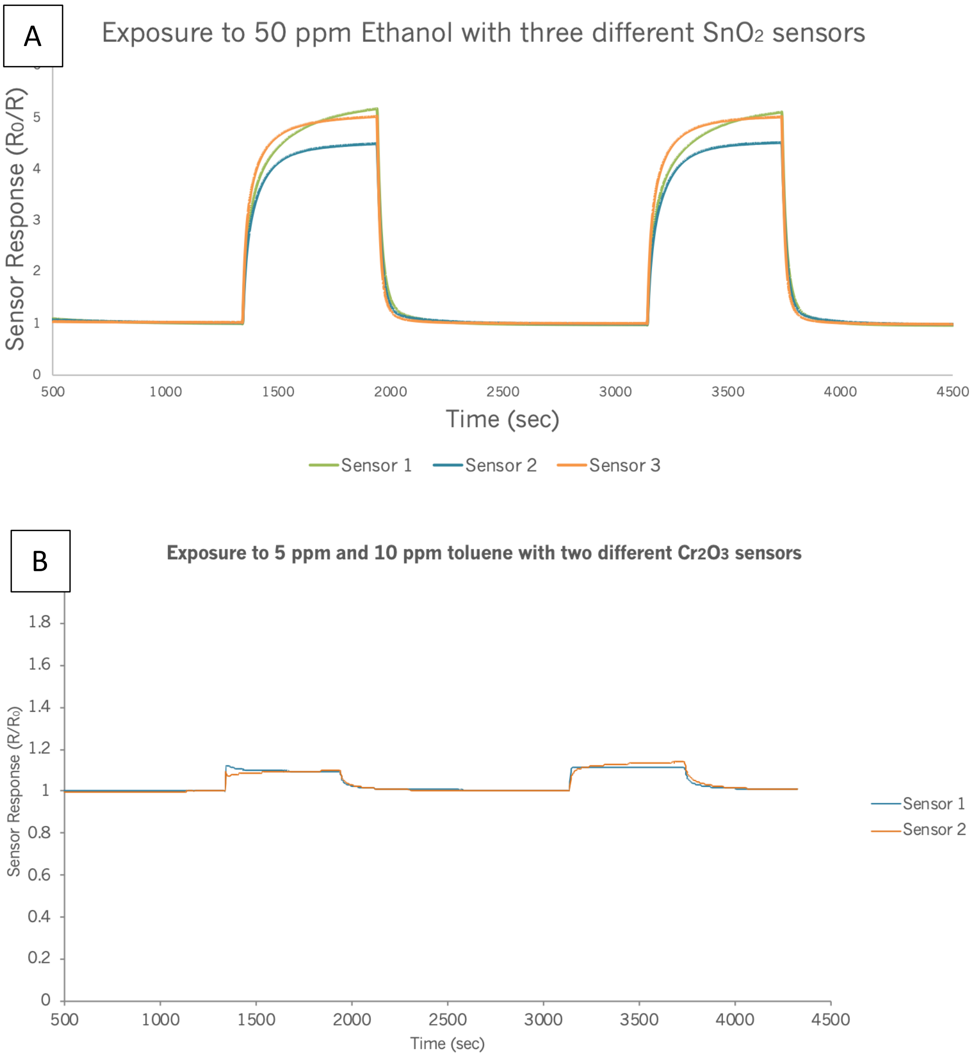

Figure S5. Sensor responses of three different SnO_2 sensors to two pulses of 50 ppm ethanol at 400 °C (A). Cr_2O_3 sensor responses to 5 and 10 ppm toluene, respectively, at 400 °C (B). This test was performed to understand repeatability from one device to another.

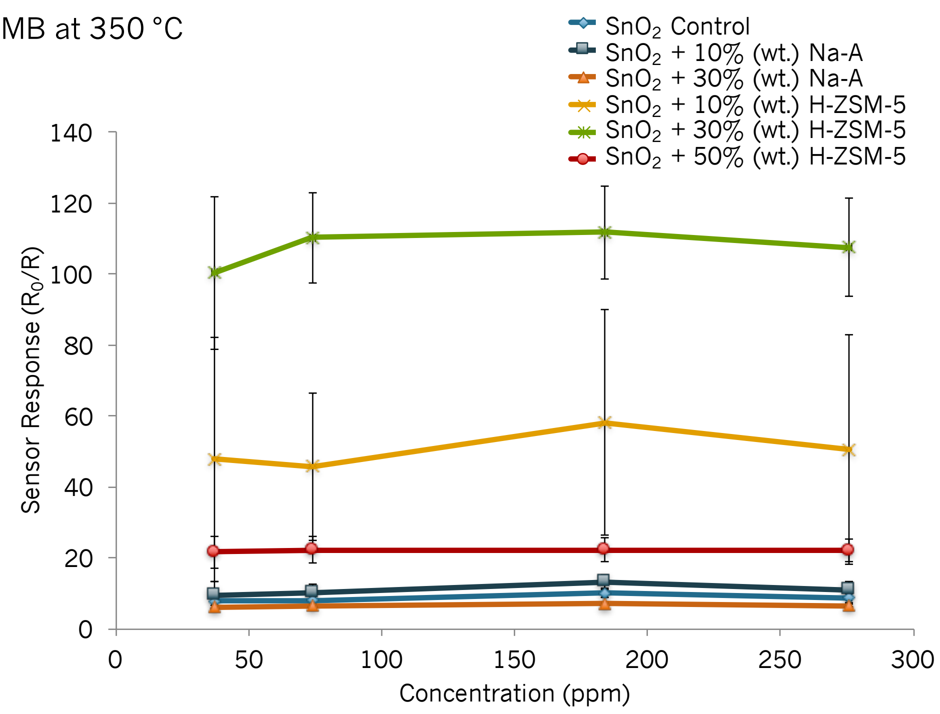

Figure S6. Sensor responses to different concentrations of methyl benzoate at 350 °C of a control SnO_2 sensor, a SnO_2 sensor modified by 10% (wt.) Na-A zeolite, another modified by 30% (wt.) Na-A, another by 10% (wt.) H-ZSM-5 and one modified by 30% (wt.) H-ZSM-5. Error bars corresponding to three repeat tests included.

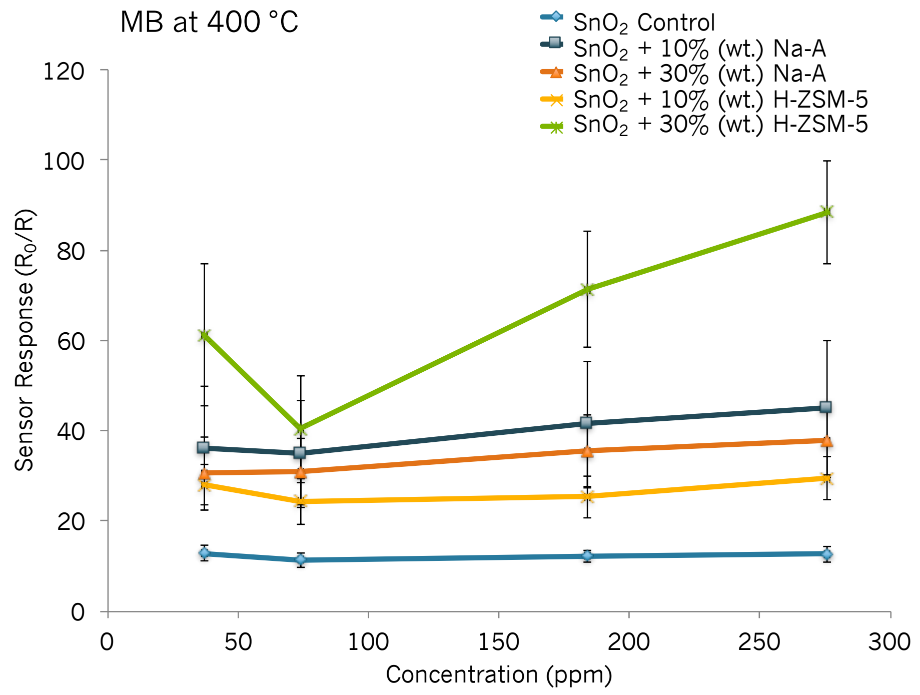

Figure S7. Sensor responses to different concentrations of methyl benzoate at 400 °C of a control SnO_2 sensor, a SnO_2 sensor modified by 10% (wt.) Na-A zeolite, another modified by 30% (wt.) Na-A, another by 10% (wt.) H-ZSM-5 and one modified by 30% (wt.) H-ZSM-5. Error bars corresponding to three repeat tests included.

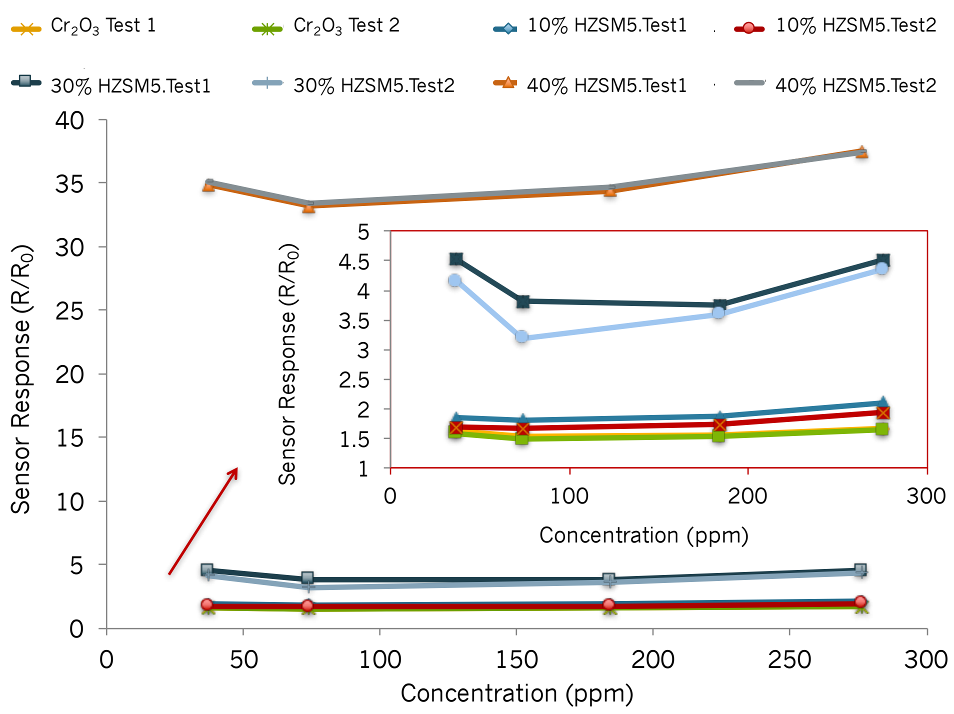

Figure S8. Sensor responses to different concentrations of MB at 350 °C of a Cr_2O_3 control sensor and those modified by 10% (wt.), 30 % (wt.) and 40% (wt.) zeolite H-ZSM-5. The inset corresponds to the lower sensor responses, which were difficult to see. The graph includes results of two repeat tests for each sensor. The inset has been provided for clarification.

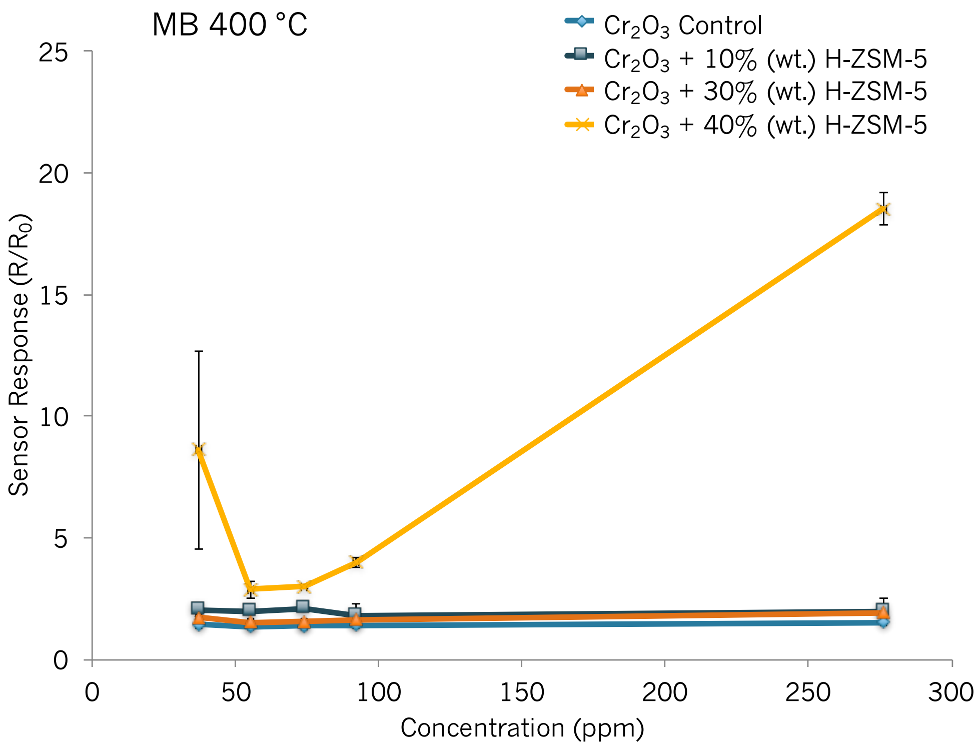

Figure S9. Sensor responses to different concentrations of MB at 400 °C of a Cr_2O_3 control sensor and those modified by 10% (wt.), 30 % (wt.) and 40% (wt.) of zeolite H-ZSM-5. The results correspond to the average of 3 repeat tests.

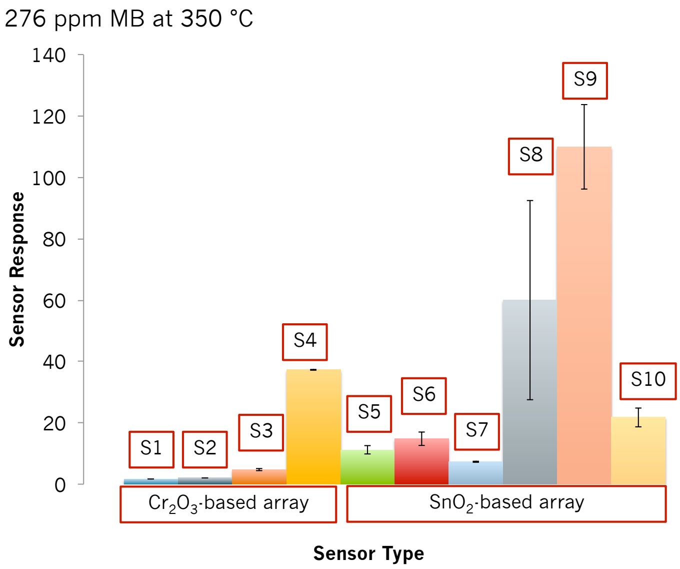

Figure S10. Sensor responses to ca. 276 ppm MB at 350 °C S1 = Control Cr_2O_3, S2 = Cr_2O_3 + 10% (wt.) H-ZSM-5, S3 = Cr_2O_3 + 30 % (wt.) H-ZSM-5, S4 = Cr_2O_3 + 40 % (wt.) H-ZSM-5, S5 = control SnO_2, S6 = SnO_2 + 10% (wt.) Na-A, S7 = SnO_2 + 30% (wt.) Na-A, S8 = SnO_2 + 10% (wt.) H-ZSM-5, S9 = SnO_2 + 30 % (wt.) H-ZSM-5, S10 = SnO_2 + 50 % (wt.) H-ZSM-5. Sensor response for p-type systems was calculated as R/R_0 and as R_0/R in n-type systems.

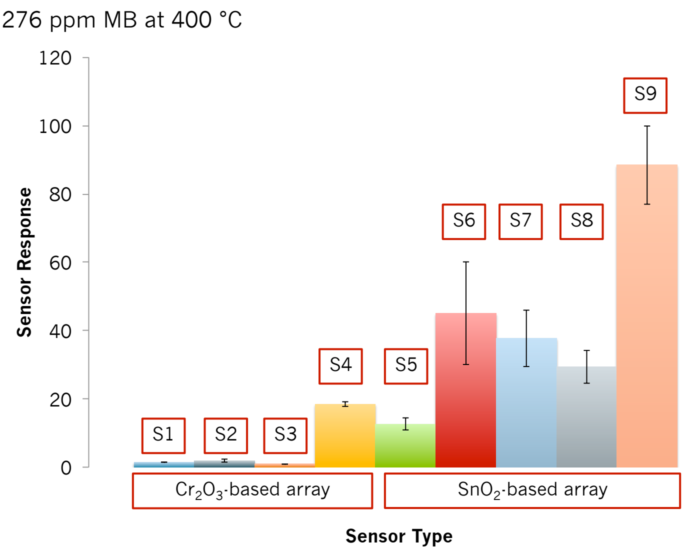

Figure S11. Sensor responses to ca. 276 ppm MB at 400 °C. S1 = Control Cr_2O_3, S2 = Cr_2O_3 + 10% (wt.) H-ZSM-5, S3 = Cr_2O_3 + 30 % (wt.) H-ZSM-5, S4 = Cr_2O_3 + 40 % (wt.) H-ZSM-5, S5 = control SnO_2, S6 = SnO_2 + 10% (wt.) Na-A, S7 = SnO_2 + 30% (wt.) Na-A, S8 = SnO_2 + 10% (wt.) H-ZSM-5, S9 = SnO_2 + 30 % (wt.) H-ZSM-5. Sensor response was calculated as R/R_0 and as R_0/R in n-type systems. Tests were repeated three times in both systems.

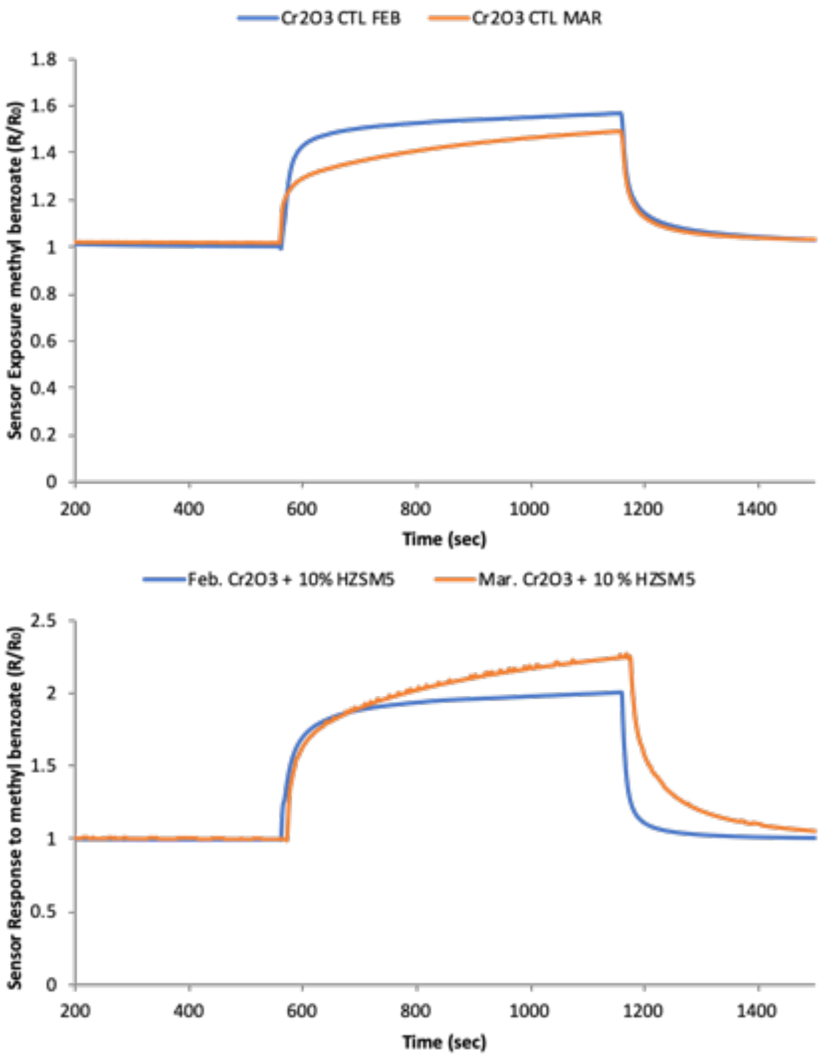

Figure S12 – Cr_2O_3-based sensor responses to 275 ppm MB after continuous use for 23 days.

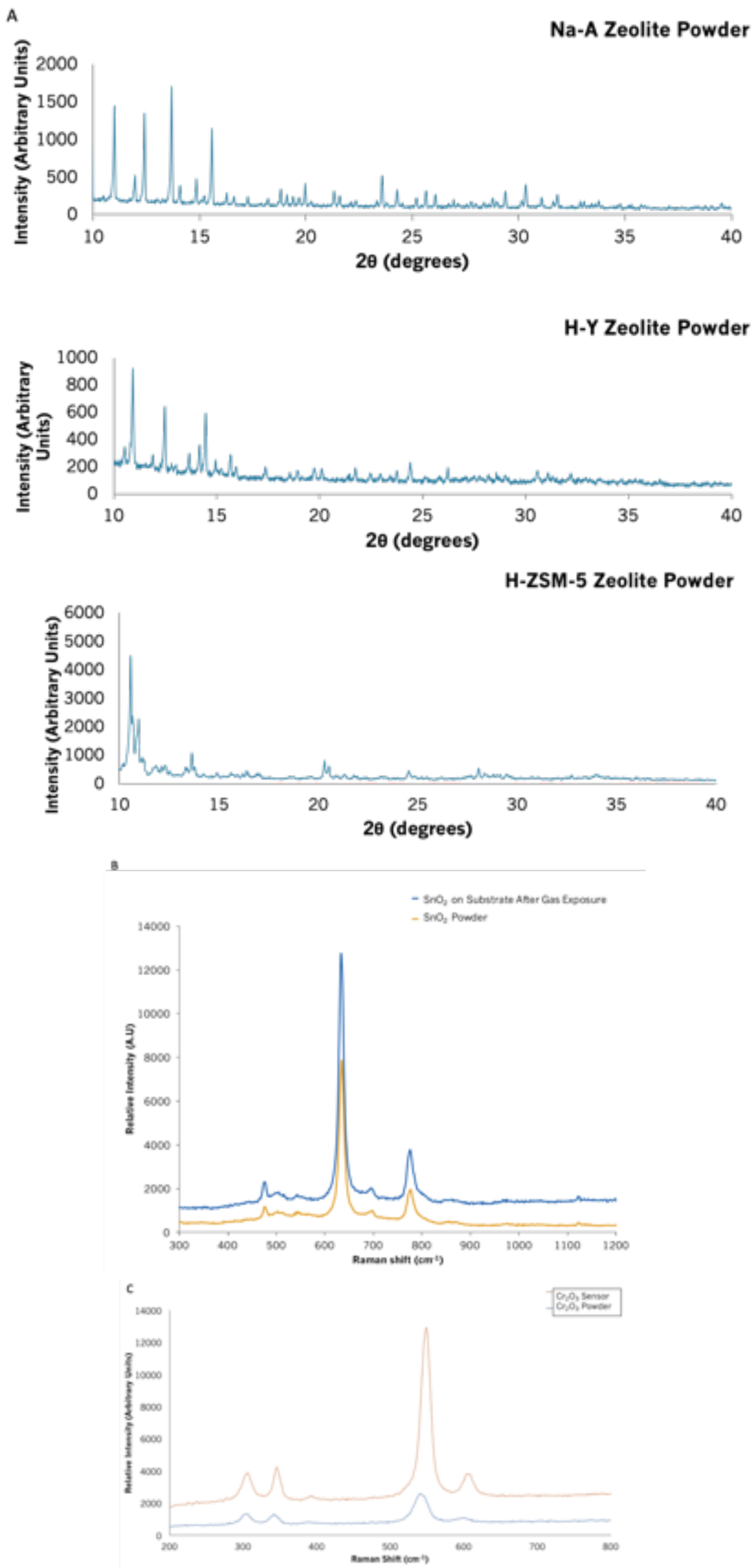

Figure S13 – Physicochemical characterization of sensing materials before and after gas sensing tests. A) XRD patterns of Na-A, H-Y, and H-ZSM-5 zeolite powders. B) Raman spectra of SnO_2 sensing material on substrate and SnO_2 powder. C) Raman spectra of Cr_2O_3 sensing material on substrate and Cr_2O_3 powder.

Support Vector Machines

The sensors were selected because they displayed selective characteristics, they also provided distinct response patterns when the response magnitudes towards some gases were similar and because variability between repeat tests was generally found to be minimal. The dataset corresponded to tests carried out at 400 °C. The input data corresponds to that attained upon sensor exposure to 9 analytes: ethanol, ethane, acetone, toluene, propane, butane, methyl benzoate, ammonia and nitrogen dioxide. Note that nitrogen dioxide was only used to compare whether the model was able to discriminate between ethane and an oxidising gas, given some sensors were previously found to provide resistive responses upon exposure to ethane.

The dataset contained information regarding the maximum conductive and maximum resistive responses of each sensor at different intervals following gas injection into the system. For instance, the response values after 5 seconds, 10 seconds, 50 seconds, 100 seconds, 200 seconds, 300 seconds, 400 seconds, 500 seconds were included initially to see how the models performed. Note that when a sensor was seen to provide high variability among repeat tests, the data was left out of the dataset.

An SVM SMO algorithm (with PolyKernel function) was used to train the dataset. The SMO algorithm offers computational speed, using a one-against-one approach. As suggested in the literature, the one-against-one approach is suitable for practical applications aimed at solving multi-class problems with a large number of training samples, as it speeds up the decision-making process and it would therefore be useful in the future as well with further data collection.(2) Kernels are used to solve multi-class problems and whilst some groups suggest that both polynomial and RBF kernel functions provide similar classification performance, other studies suggest that the performance of the polynomial kernel is consistently better when dealing with a large number of attributes. The classification performance of random forests has previously been reported as comparable to that of SVMs.(3) For this reason, the classification performance of random forests was also evaluated to understand the robustness of the SVMs in accurately classifying the data into gas type.

Table S1 – Gas concentrations (in ppm) investigated in this study and used as data for the SVMs. MB refers to methyl benzoate.

Gas	Toluene	Nitrogen Dioxide	Butane	Ethanol	Ethane	Propane	Ammonia	MB	Acetone
Concentration (ppm)	5	0.05	10	10	10	10	5	37	0.5
	10	0.1	20	20	20	20	10	55	1
	25	0.2	50	50	50	50	25	74	2
	40	0.5	80	80	80	80	40	92	5
	50	0.8	100	100	100	100	50	184	8
								276	10
									20
									50
									80
									100

Table S2 – Summary table of all sensors used in this study. Two different concentrations and two different temperatures have been included for illustration purposes. Desorption was assessed according to the peak shapes. Peak tailing is directly related to baseline drift and sensor recovery. T_{90} and T_{10} values have been included for sensor heating temperatures of 400 °C, which was the temperature employed for the SVMs. Cells marks with a tick means 'Yes' and with a cross 'No'. Cells marked with '~' mean 'slight'.

	Concentration (ppm)	350 °C					400 °C				
**n-type**		R_{max}	**Steady State**	**Tailing**	T_{90} (sec)	T_{10} (sec)	R_{max}	**MB Desorption**	**Tailing**	T_{90} (sec)	T_{10} (sec)
**SnO_2**	37	7.8 ± 2.4	✓	✗			12.8 ± 1.7	✓	✗		
**SnO_2 + 10% wt. Na-A**	37	9.5 ± 3.8	✓	~			36.1 ± 13.8	✓	✗		
**SnO_2 + 30% wt. Na-A**	37	6.0 ± 0.7	✓	~			30.5 ± 8.1	✓	✓		
**SnO_2 + 10% wt. H-ZSM-5**	37	47.7 ± 34.4	✓	✗			27.9 ± 4.5	✓	✗		
**SnO_2 + 30% wt. H-ZSM-5**	37	100.4 ± 21.5	✗	✓			61.1 ± 15.7	✓	✗		
**SnO_2 + 50% wt. H-ZSM-5**	37	21.6 ± 4.6	✗	✓			203.1 ± 73.3	✓	✓		
**SnO_2**	276	11.1 ± 1.4	✗	✗	10	516	12.68 ± 1.72	✓	✗	2	282
**SnO_2 + 10% wt. Na-A**	276	14.8 ± 2.2	✗	✗	8	910	45.1 ± 14.9	✗	✗	2	892
**SnO_2 + 30% wt. Na-A**	276	7.3 ± 0.27	✓	✗	14	945	37.8 ± 8.3	✓	✓	2	937
**SnO_2 + 10% wt. H-ZSM-5**	276	60.1 ± 32.5	✗	✗	2	653	29.5 ± 4.9	✓	✗	2	361
**SnO_2 + 30% wt. H-ZSM-5**	276	110.0 ± 13.8	✗	✓	2	535	88.4 ± 11.5	✗	✗	2	716
**SnO_2 + 50% wt. H-ZSM-5**	276	21.8 ± 3.2	✗	~	4	806	247.3 ± 80.6	✗	✗	2	861
**p-type**	**Concentration (ppm)**	R_{max}	**Desorption**	**BL Drift**	T_{90} (sec)	T_{90} (sec)	R_{max}	**MB Desorption**	**BL Drift**	T_{90} (sec)	T_{10} (sec)
**Cr_2O_3**	37	1.6	✓	✗			1.4 ± 0.0	✓	✗		
**Cr_2O_3 + 10% wt. H-ZSM-5**	37	1.7	✓	✗			2.0 ± 0.1	✓	✗		
**Cr_2O_3 + 30% wt. H-ZSM-5**	37	4.1	✓	✓			1.7 ± 0.1	✓	✗		
**Cr_2O_3 + 40% wt. H-ZSM-5**	37	35.1	✗	✓			8.6 ± 4.1	✓	✗		
**Cr_2O_3 + H-Y overlays**	37	12.3	✗	✓			13.1 ± 0.1	✓	✓		
**Cr_2O_3**	276	1.6	✓	✗	347	200	1.5 ± 0.0	✓	✗	357	112
**Cr_2O_3 + 10% wt. H-ZSM-5**	276	1.9	✓	✗	343	875	2.0 ± 0.5	✓	✗	361	190
**Cr_2O_3 + 30% wt. H-ZSM-5**	276	4.4	✓	✓	396	135	1.9 ± 0.0	✓	✗	410	59
**Cr_2O_3 + 40% wt. H-ZSM-5**	276	37.4	✗	✓	345	84	18.5 ± 0.7	✗	✗	512	10
**Cr_2O_3 + H-Y overlays**	276	13.6	✗	✓			14.7 ± 0.5	✗	✓	312	70

References:

1. Kim JH, Jeong HM, Na CW, Yoon JW, Abdel-Hady F, Wazzan AA, et al. Highly selective and sensitive xylene sensors using Cr2O3-ZnCr2O4 hetero-nanostructures prepared by galvanic replacement. Sensors Actuators, B Chem [Internet]. 2016;235:498–506. Available from: http://dx.doi.org/10.1016/j.snb.2016.05.104
2. Milgram J, Cheriet M, Sabourin R. “One Against One” or “One Against All”: Which One is Better for Handwriting Recognition with SVMs? Tenth Int Work Front Handwrit Recognit [Internet]. 2006;1–6. Available from: http://hal.inria.fr/inria-00103955
3. Caruana R, Niculescu-Mizil A. An empirical comparison of supervised learning algorithms. Proc 23rd Int Conf Mach Learn [Internet]. 2006;C(1):161–8. Available from: http://portal.acm.org/citation.cfm?doid=1143844.1143865
